# The Etiology of Pneumonia in Zambian Children: Findings From the Pneumonia Etiology Research for Child Health (PERCH) Study

**DOI:** 10.1097/INF.0000000000002652

**Published:** 2021-08-25

**Authors:** Lawrence Mwananyanda, Donald M. Thea, James Chipeta, Geoffrey Kwenda, Justin M. Mulindwa, Musaku Mwenechanya, Christine Prosperi, Melissa M. Higdon, Meredith Haddix, Laura L. Hammitt, Daniel R. Feikin, David R. Murdoch, Katherine L. O’Brien, Maria Deloria Knoll, James Mwansa, Somwe Wa Somwe, Phil Seidenberg

**Affiliations:** From the * Department of Global Health, Boston University School of Public Health, Boston, Massachusetts; † Right To Care-Zambia, Lusaka, Zambia; ‡ Department of Paediatrics and Child Health, University of Zambia School of Medicine, Lusaka, Zambia; § Department of Paediatrics, University Teaching Hospital, Lusaka, Zambia; ¶ Department of Biomedical Sciences, School of Health Sciences, University of Zambia, Lusaka, Zambia; ‖ Department of International Health, International Vaccine Access Center, Johns Hopkins Bloomberg School of Public Health, Baltimore, Maryland; ** Department of Pathology and Biomedical Sciences, University of Otago, Christchurch, New Zealand; †† Microbiology Unit, Canterbury Health Laboratories, Christchurch, New Zealand; ‡‡ Department of Pathology and Microbiology, University Teaching Hospital, Lusaka, Zambia; §§ Department of Microbiology, Lusaka Apex Medical University, Lusaka, Zambia; ¶¶ Department of Emergency Medicine, University of New Mexico, Albuquerque, New Mexico.

**Keywords:** Zambia, pneumonia, etiology, child, Pneumonia Etiology Research for Child Health

## Abstract

Childhood pneumonia in developing countries is the foremost cause of morbidity and death. Fresh information on etiology is needed, considering the changing epidemiology of pneumonia in the setting of greater availability of effective vaccines, changing antibiotic use and improved access to care. We report here the Zambia site results of the Pneumonia Etiology Research for Child Health study on the etiology of pneumonia among HIV-uninfected children in Lusaka, Zambia.

**Methods:**

We conducted a case–control study of HIV-uninfected children age 1–59 months admitted with World Health Organization-defined severe or very severe pneumonia to a large tertiary care hospital in Lusaka. History, physical examination, chest radiographs (CXRs), blood cultures and nasopharyngeal/oropharyngeal swabs were obtained and tested by polymerase chain reaction and routine microbiology for the presence of 30 bacteria and viruses. From age and seasonally matched controls, we tested blood and nasopharyngeal/oropharyngeal samples. We used the Pneumonia Etiology Research for Child Health integrated analysis to determine the individual and population etiologic fraction for individual pathogens as the cause of pneumonia.

**Results:**

Among the 514 HIV-uninfected case children, 208 (40.5%) had abnormal CXRs (61 of 514 children were missing CXR), 8 (3.8%) of which had positive blood cultures. The overall mortality was 16.0% (82 deaths). The etiologic fraction was highest for respiratory syncytial virus [26.1%, 95% credible interval (CrI): 17.0–37.7], *Mycobacterium tuberculosis* (12.8%, 95% CrI: 4.3–25.3) and human metapneumovirus (12.8%, CrI: 6.1–21.8).

**Conclusions:**

Childhood pneumonia in Zambia among HIV-uninfected children is most frequently caused by respiratory syncytial virus, *M. tuberculosis* and human metapneumovirus, and the mortality remains high.

## Abstract 2

Supplemental Digital Content is available in the text.

Despite globally improved child and infant mortality rates, many low- and middle-income countries have not yet achieved the Millennium Development Goal 4 target for under-5 mortality.^1^ Much of this is due to pneumonia which remains the leading infectious cause of death among children under 5 years in developing countries.^2,3^ Efforts to reduce under-5 pneumonia deaths include improved case management and availability of highly effective respiratory vaccines. Despite these efforts, approximately 6000 under-5 children die of pneumonia every year in Zambia, comprising 15% of all deaths in this age group.^4^

To improve pneumonia outcomes further in Zambia, we need a more complete understanding of risk factors, and the microbiologic causes of pneumonia, specific to the local setting. A Zambian site was included in the Pneumonia Etiology Research for Child Health (PERCH) study, described in detail elsewhere.^5,6^ Briefly, PERCH is a highly standardized 7-country case–control study of under-5 children hospitalized with World Health Organization (WHO) defined severe and very severe pneumonia (pre-2013), with a primary aim to determine the microbiologic etiology of pneumonia and risk factors for severe and very severe pneumonia.

This report is limited to the findings from the Zambia site, which is typical of the majority of countries in Eastern and Southern Africa with regard to community or environmental pneumonia risk factors. Because risk factors and etiology are likely to differ by HIV status, we restrict this analysis to the etiology of pneumonia among HIV-negative children enrolled at the Zambia site. Findings among the HIV-infected children are reported separately in this issue.^7^

## MATERIALS AND METHODS

### Location

Zambia is a landlocked middle-income country in Southern Africa with relatively high infant mortality (56 per 1000) and HIV prevalence (19.4% among Lusaka women of child-bearing age).^8^ Childhood vaccines are widely available and distributed, although pneumococcal conjugate vaccine (PCV) was not introduced until the end of the study. Detailed country characteristics are available in Supplemental Digital Content 1, http://links.lww.com/INF/D850. This study was conducted at the University Teaching Hospital (UTH) in Lusaka, the densely populated capital of Zambia.^9^ UTH is a 1500-bed academic and tertiary healthcare facility in Lusaka serving the greater Lusaka district, including the most impoverished segments of the population, and is a referral center for the entire country. Access to mechanical ventilation was limited and rarely used. Obtaining radiographs required taking children to the Radiology Department at some distance from the pediatric wards and at the caregivers’ expense, therefore, were not routinely performed (for nonstudy patients). Oxygen, however, was routinely available. Nearly all children presenting to UTH are initially seen and evaluated at surrounding Lusaka clinics, transported (mostly by private means) to UTH and have often received initial antibiotics (typically benzylpenicillin G) from the clinic, if clinically indicated.

### Participants

Study participants were children 1–59 months of age living within the Lusaka catchment area. Details of study methods have been published elsewhere, and are described in Supplemental Digital Content 1, http://links.lww.com/INF/D850.^5,10^ Briefly, cases were children hospitalized between November 2011 and October 2013 with WHO-defined^11^ severe or very severe pneumonia (pre-2013), including cough and/or difficulty in breathing plus danger signs (central cyanosis, difficulty breast-feeding/drinking, vomiting everything, multiple or prolonged convulsions, lethargy/unconsciousness, or head nodding) defined as “very severe pneumonia,” or lower chest wall in-drawing in the absence of danger signs defined as “severe pneumonia.” Due to constraints in processing samples on weekends and study staffing, enrolment occurred on weekdays from 07:30 to 18:00 hours. Nighttime admissions were eligible for participation the following morning.

Controls were children without case-defining pneumonia who were randomly selected from the community and frequency matched on age and season (within 3 weeks) to cases. They were not excluded if they had evidence of upper respiratory tract infections.

Cases and controls were excluded if they had been hospitalized within the previous 14 days or if they were a PERCH study participant within 30 days; cases were also excluded if lower chest wall in-drawing resolved with bronchodilator challenge (if applicable).

### Clinical Procedures

Standardized study clinical examinations for cases occurred on admission and at 24 and 48 hours. One-month postdischarge, examinations occurred to determine clinical and vital status. Chest radiographs (CXR) were obtained at admission from cases and classified as normal, consolidation, other infiltrate, consolidation and other infiltrate or uninterpretable as defined by the WHO standardized interpretation of pediatric CXRs.^12,13^ For controls, clinical assessments only occurred at time of enrolment in PERCH.

### Specimen Collection and Laboratory Methods

Specimen collection, microbiologic testing and laboratory methods were highly standardized (Supplemental Digital Content 1, http://links.lww.com/INF/D850).^14–20^ Blood and nasopharyngeal/oropharyngeal (NP/OP) specimens were collected from cases and controls. Induced sputum and pleural fluid specimens (where clinically indicated) were collected from cases only for culture and polymerase chain reaction (PCR) testing. Positivity was defined using quantitative PCR density thresholds for four pathogens where there was similar prevalence in cases and controls; these include *Streptococcus pneumoniae* (≥2.2 log10 copies/mL) from whole blood^17^ and *S. pneumoniae* (≥6.9 log10 copies/mL),^21^ *Haemophilus influenzae* (≥5.9 log10 copies/mL),^19^ cytomegalovirus (CMV, ≥4.9 log10 copies/mL) and Pj (≥4 log10 copies/mL),^19^ NP/OP (CMV threshold analysis available from authors).

HIV exposure status of the child was obtained from the mother’s health antenatal card or by maternal recall if card not available. All exposed children had HIV testing performed by DNA PCR or serology, as per Zambian National HIV Guidelines.

### Statistical Analysis

Odds ratios and 95% confidence intervals (CIs) of pathogens detected on NP/OP PCR in cases compared with controls were calculated using logistic regression adjusted for age in months and presence of all other pathogens detected on NP/OP PCR to account for associations between pathogens. Logistic regression adjusted for age in months was used to compare clinical characteristics by case–control status and, among cases, by vital status. Results were stratified by age, severity, CXR status and mortality. *P* values <0.05 were considered significant.

The percent of pneumonia due to each pathogen was estimated using the PERCH integrated analysis (PIA) method, which is described in detail elsewhere.^22–24^ In brief, the PIA is a Bayesian nested partially latent class analysis that integrates the results for each case from blood culture, NP/OP PCR, whole blood PCR for pneumococcus and induced sputum culture for *Mycobacterium tuberculosis*. The PIA also integrates test results from controls to account for imperfect test specificity of NP/OP PCR and whole blood PCR. Blood culture results (excluding contaminants) and induced sputum or gastric aspirate results for *M. tuberculosis* were assumed to be 100% specific (ie, the etiology for a case was attributed 100% to the pathogen that was detected in their blood by culture).

The PIA accounts for imperfect sensitivity of each test/pathogen measurement by using a priori estimates of their sensitivity (ie, estimates regarding the plausibility range of sensitivity which varied by laboratory test method and pathogen). Sensitivity of blood culture was reduced if blood volume was low (<1.5 mL) or if antibiotics were administered before specimen collection. Sensitivity of NP/OP PCR for *S. pneumoniae* and *H. influenzae* was reduced if antibiotics were administered before specimen collection (Supplemental Digital Content 2, http://links.lww.com/INF/D851).

As a Bayesian analysis, both the list of pathogens and their initial “prior” etiologic fraction (EF) values were specified a priori, which favored no pathogen over another (ie, “uniform”). The pathogens selected for inclusion in the analysis included any noncontaminant bacteria detected by culture in blood at any of the 9 PERCH sites, regardless of whether it was observed at the Zambia site specifically, *Mycobacterium tuberculosis*, and all of the multiplex quantitative PCR pathogens except those considered invalid because of poor assay specificity (*Klebsiella pneumoniae*^25^ and *Moraxella catarrhalis*). A category called “Pathogens Not Otherwise Specified” was also included to estimate the fraction of pneumonia caused by pathogens not tested for or not observed. A child negative for all pathogens would still be assigned an etiology, which would be either one of the explicitly estimated pathogens (implying a “false negative,” accounting for imperfect sensitivity of certain measurements) or not otherwise specified. The model assumes that each child’s pneumonia was caused by a single pathogen.

Analyses were adjusted for age (<1 vs. ≥1 year) to account for differences in pathogen prevalences by this factor; analyses stratified by pneumonia severity could not adjust for age due to small sample size. For results stratified by case clinical data (eg, to CXR+, very severe, vital status, etc.), the test results from all controls were used. However, for analyses stratified by age, only data from controls representative of that age group were used. A separate integrated etiology model was run with in-hospital vital status as a covariate, adjusting for the child’s age.

The PIA estimated both the individual- and population-level etiology probability distributions, each summing to 100% across pathogens where each pathogen has a probability ranging from 0% to 100%. The population-level EF estimate for each pathogen was approximately the average of the individual case probabilities and was provided with a 95% credible interval (95% CrI), the Bayesian analogue of the CI.

Statistical analyses were conducted using SAS 9.3 (SAS Institute, Cary, NC), R Statistical Software 3.3.1 (The R Development Core Team, Vienna, Austria) and Bayesian inference software JAGS 4.2.0 (http://mcmc-jags.sourceforge.net/). The R package used to perform the PIA, named the Bayesian Analysis Kit for Etiology Research, is publicly available at https://github.com/zhenkewu/baker.

### Ethical Considerations

The study protocol was approved by the Institutional Review Boards at Boston University, the Johns Hopkins Bloomberg School of Public Health in the United States and by the ERES Converge Ethical Review Committee in Zambia. Parents or guardians of participants provided written informed consent.

## RESULTS

### Demographics and Clinical Characteristics

**FIGURE 1.**
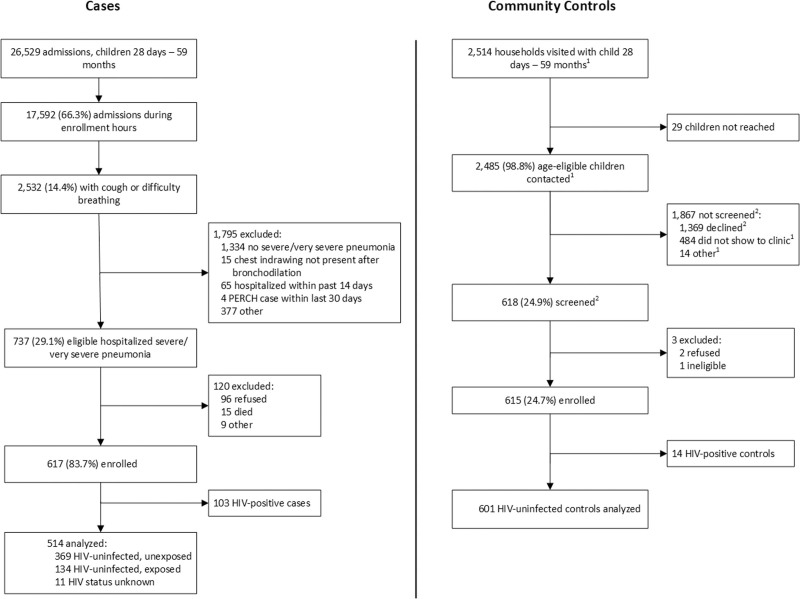
Case and Control enrollment flow diagram. 1. Numbers estimated for 4 months. 2. Numbers estimated for 9 months. Number of children not screened may include children who declined to be screened/were not able to be located before age eligibility could be determined.

Among 17,592 children 1–59 months of age admitted to the Pediatric Wards at UTH during the study period, 737 (4.1%) met the case definition of WHO severe or very severe pneumonia (Fig. 1). Six hundred seventeen (83.7%) of these consented and were enrolled. This report is limited to the 514 (83.3%) HIV-uninfected cases (severe: 354, 68.9%; very severe: 160, 31.1%); clinical and etiology details of the remaining 103 (16.7%) HIV-infected cases will be reported elsewhere. Six hundred one HIV-uninfected controls were consented and enrolled from Lusaka communities within UTH catchment and are included here (Fig. 1). A small proportion (69/601, 11.5%) of the controls had evidence of upper respiratory tract infections at the time of enrolment (Table 1). There was some seasonal variation in disease—particularly with respiratory syncytial virus (RSV) (Supplemental Digital Content 3, http://links.lww.com/INF/D852), with a greater number of cases (313, 60.9% of total) being enrolled during the Zambia rainy period (November to May).

**TABLE 1.** Demographic and Clinical Characteristics of HIV-uninfected Cases With Severe and Very Severe Pneumonia on Admission and Controls Demographic and Clinical Characteristics of HIV-uninfected Cases and Controls

Characteristics	All Cases	CXR+ Cases	Controls
All	514	208	601
Age			
Median age in months (IQR)	5 (2–11)	5 (2–11)	6 (3–12)
1–5 months	276 (53.7)	110 (52.9)	286 (47.6)
6–11 months	123 (23.9)	49 (23.6)	150 (25.0)
12–23 months	76 (14.8)	37 (17.8)	108 (18.0)
24–59 months	39 (7.6)	12 (5.8)	57 (9.5)
Sex			
Male	280 (54.5)	117 (56.3)	301 (50.1)
Female	234 (45.5)	91 (43.8)	300 (49.9)
HIV exposure status			
HIV exposed/uninfected (HIV exposed)	134 (26.1)	55 (26.4)	168 (28.0)
HIV unexposed/uninfected	369 (71.8)	151 (72.6)	422 (70.2)
Unknown	11 (2.1)	2 (1.0)	11 (1.8)
Respiratory tract illness (controls only) *	—	—	69 (11.5)
DTP/Hib fully vaccinated for age†			
<1 year old	288 (75.6)	113 (74.8)	368 (84.8)
≥1 year old	91 (92.9)	40 (95.2)	152 (92.1)
Total	379 (79.1)	153 (79.3)	520 (86.8)
At least 1 dose of measles vaccine‡	94 (83.9)	40 (85.1)	167 (88.8)
Weight-for-age (WHO) Z scores			
>−2 Z scores	353 (68.8)	142 (68.3)	525 (87.5)
≤−2 Z scores	160 (31.2)	66 (31.7)	75 (12.5)
Antibiotic pretreatment before specimen collection§	462 (91.5)	189 (92.2)	93 (15.9)
Serum antibiotic activity	141 (28.8)	50 (25.3)	21 (3.9)
Very severe pneumonia	160 (31.1)	70 (33.7)	—
CXR available	453 (88.1)	208 (100)	—
CXR result			—
Any consolidation	135 (29.8)	135 (64.9)	—
Other infiltrate only	73 (16.1)	73 (35.1)	—
Normal	159 (35.1)	0 (0.0)	—
Uninterpretable	86 (19.0)	0 (0.0)	—
Median duration of illness¶ in days (IQR)	3 (2, 5)	3 (2, 6)	—
Duration of illness at enrolment¶			—
0–2 d	181 (35.4)	64 (30.8)	—
3–5 d	220 (43.1)	91 (43.8)	—
>5 d	110 (21.5)	53 (25.5)	—
Median duration of hospitalization in days (IQR)	4 (2, 7)	5 (3, 8)	—
Hypoxemia‖	185 (36.1)	97 (46.6)	—
Tachypnea**	443 (87.0)	188 (90.8)	—
Tachycardia††	328 (64.6)	135 (65.2)	—
Head nodding	69 (13.4)	31 (14.9)	—
Central cyanosis	18 (3.5)	8 (3.8)	—
Convulsions	25 (4.9)	9 (4.3)	—
Lethargy	64 (12.5)	28 (13.5)	—
Unable feed	44 (8.6)	16 (7.7)	—
Wheeze on auscultation	63 (12.3)	22 (10.6)	—
Grunting	137 (26.7)	56 (26.9)	—
Elevated temperature (≥38°C)	267 (52.1)	122 (58.9)	—
Leukocytosis‡‡	213 (42.7)	97 (47.8)	—
CRP ≥ 40 mg/L	165 (35.6)	91 (48.4)	—
Severe anemia§§	41 (8.2)	18 (8.9)	—
Died in hospital or within 30 days of admission	82 (16.0)	25 (12.0)	—
Died in hospital	76 (14.8)	23 (11.1)	—
Died within 24 hours of admission	37 (7.2)	6 (2.9)	—
Died postdischarge, within 30 days of admission¶¶	6 (2.7)	2 (1.9)	—
Died within 7 days of discharge¶¶	2 (0.9)	1 (1.0)	—
Missing 30-day vital status	216 (42.0)	82 (39.4)	—

bpm indicates beats per minute; CRP, C-reactive protein; DTP, diphtheria-tetanus-pertussis vaccine; IQR, interquartile range.

*Respiratory tract illness was defined as presence of cough or runny nose, or if a child had (1) at least 1 of ear discharge, wheezing or difficulty breathing and (2) either a measured temperature of >38.0°C within the previous 48 hours or a history of sore throat.

†Pentavalent vaccine (DTP-Hib-HepB) used in Zambia. For children <1 year, defined as received at least 1 dose and up-to-date for age based on the child’s age at enrollment, doses received and country schedule (allowing 4-week window each for dose). For children >1 year, defined as ≥3 doses. Restricted to those with available Pentavalent vaccine data.

‡Restricted to those children >10 months of age with available measles vaccine data.

§Defined as serum bioassay positive (cases and controls), antibiotics administered at the referral facility, or antibiotic administration before whole blood specimen collection at the study facility (cases only).

¶Duration of illness defined as duration (in days) of cough, wheeze, fever or difficulty breathing, whichever is longest.

‖Hypoxemia defined as oxygen saturation <90% or on supplemental oxygen if a room air oxygen saturation reading was not available.

**Tachypnea defined as ≥60 breaths/min (<2 months), ≥50 breaths/min (2–11 months) and ≥40 breaths/min (12–59 months).

††Tachycardia defined as >160 bpm (0–11 months), >150 bpm (12–35 months), >140 bpm (36–59 months).

‡‡Leukocytosis defined as >15 × 10^9^ cells/L for children 1–11 months and >13 × 10^9^ cells/L for children 12–59 months.

§§Defined as hemoglobin 0–7.5 g/dL.

¶¶Restricted to children discharged alive who had vital status data obtained ≥21 days following admission.

The median case age was 5 months (vs. 6 months for controls), and 77.6% were infants <1 year of age (Table 1). The percentage of children who were HIV-exposed (ie, mother HIV infected at delivery) but uninfected was similar between cases (26.1%) and community controls (28.0%).

Both cases and controls were up-to-date for *Haemophilus influenzae* type b vaccination (79.1% and 86.8%, respectively). Among cases, 31.2% were moderately to severely malnourished, measuring ≤−2 Z scores in WHO weight-for-age calculations. Owing to referral practices for children seen at UTH, almost all cases (91.5%) received a dose of antibiotics at the referring clinic before enrolment and specimen collection.

### Clinical Description of Cases

Interpretable CXR findings were available for 367 of 514 (71.4%) cases; of these, 208 (56.7%) showed evidence of either consolidation or “other infiltrate” (Table 1). Sixty-one cases did not have a CXR obtained due to early death (n = 30) or technical reasons (n = 31). An additional 86 were uninterpretable. CXR+ cases had longer duration of illness at enrollment and were more likely to present with hypoxia, fever, leukocytosis and elevated C-reactive protein (Supplemental Digital Content 4, http://links.lww.com/INF/D853) than CXR normal cases (*P* < 0.05). Compared with severe pneumonia cases, very severe children were more likely to be hypoxic, severely anemic and be wheezing on auscultation (*P* < 0.05) (Supplemental Digital Content 4, http://links.lww.com/INF/D853).

### Specimen Microbiology

Blood cultures were obtained from 98.2% of cases, with a pathogen identified in 24 (4.8%) (Supplemental Digital Content 5, http://links.lww.com/INF/D854) cases overall, but only in 8 (3.9%) of those included in the etiology analysis (CXR+ cases). In descending order, the frequency of pathogen isolation were *Salmonella* (n = 7, 6 nontyphoidal), *H. influenzae* (n = 5, 3 nontype b), *Staphylococcus aureus* (n = 4), *Escherichia coli* (n = 4,), *S. pneumoniae* (n = 3, all PCV10 vaccine type) and *Candida albicans* (n = 1). Bacteremia was more common among children who died in hospital (8/69, 11.6%) compared with those who survived to discharge (16/436, 3.7%). Two cases had pleural fluid specimens obtained; both were positive for *S. aureus* on culture, and one was also positive for bocavirus by PCR (data not shown).

Malaria was infrequently detected at our Zambia site, with only 1 case (0.2%) and 5 controls (0.8%) testing positive by rapid diagnostic testing and thus not included as a potential cause in the integrated etiology analysis.

*Mycobacterium tuberculosis* was isolated from induced sputum in 5 (1.2%) cases (Supplemental Digital Content 5, http://links.lww.com/INF/D854), all of whom were under 11 months of age and had abnormal CXR findings (2.7% of CXR+ cases).

More than 90% of children had a pathogen detected on NP/OP PCR (Supplemental Digital Content 6, http://links.lww.com/INF/D855, Supplemental Digital Content 7, http://links.lww.com/INF/D856). Pathogens associated with CXR+ case status on NP/OP PCR included RSV, parainfluenza 3, influenza A, human metapneumovirus (HMPV) A/B, *Pneumocystis jirovecii*, and *H. influenzae*, and *S. pneumoniae* by whole blood PCR. *P. jirovecii* was more common among HIV-uninfected exposed cases (11.2%) compared with HIV-uninfected unexposed cases (4.3%), with similar prevalence among controls by HIV exposure status (data not shown).

### Etiologic Distribution of Severe/Very Severe Pneumonia

**FIGURE 2.**
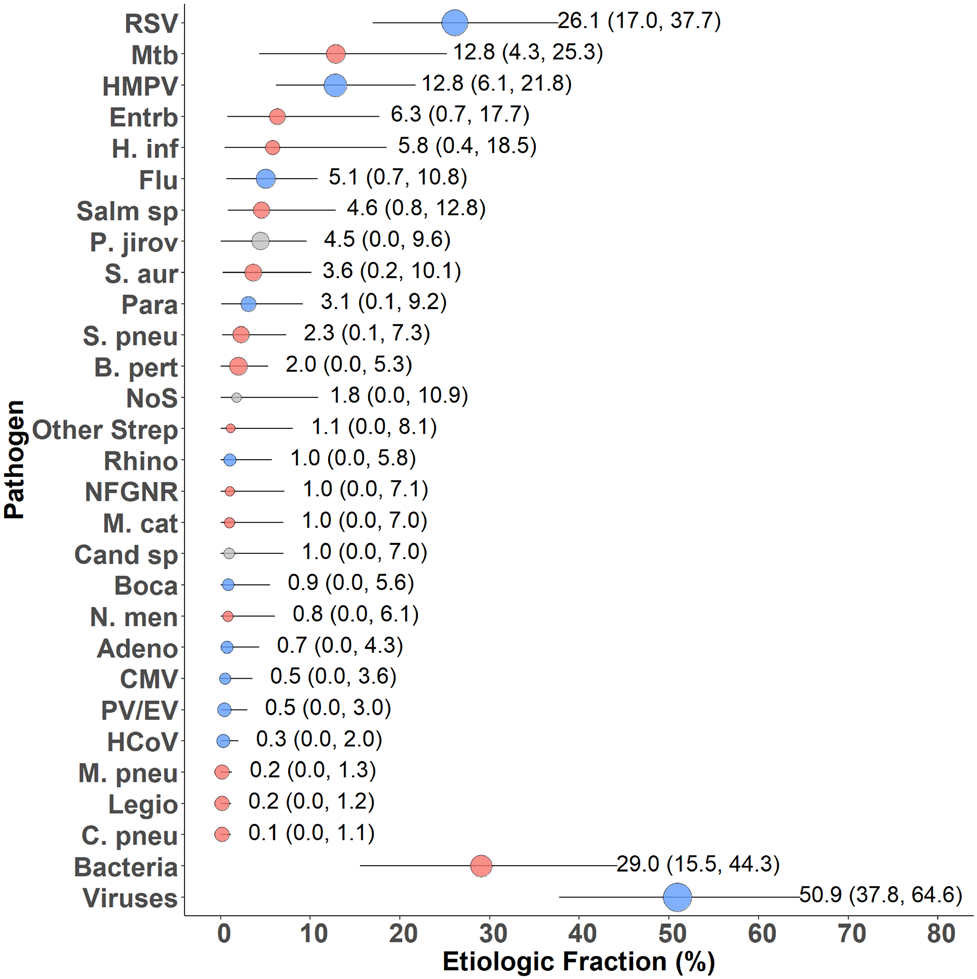
Etiologic fraction of pneumonia among HIV-uninfected cases of pneumonia with positive finding on chest radiograph. Other Strep includes *Streptococcus pyogenes* and *Enterococcus faecium*. NFGNR includes Acinetobacter species and Pseudomonas species. Enterobacteriaceae includes *E. coli*, Enterobacter species, and Klebsiella species, excluding mixed gram-negative rods. Positive finding defined as consolidation and/or other infiltrate on chest radiograph. Bacterial summary excludes Mtb. Analysis adjusted for age. Pathogens estimated at the subspecies level but grouped to the species level for display (Parainfluenza virus type 1, 2, 3 and 4; *S. pneumoniae* PCV10 and *S. pneumoniae* non-PCV10 types; *H. influenzae* type b and *H. influenzae* non-b; influenza A, B, and C). Exact figures and subspecies and serotype disaggregation (eg, PCV10 type and non-PCV10 type) are given in Supplemental Digital Content 8, http://links.lww.com/INF/D857. Description of symbols: Line represents the 95% credible interval. The size of the symbol is scaled based on the ratio of the estimated etiologic fraction to its standard error. Of 2 identical etiologic fraction estimates, the estimate associated with a larger symbol is more informed by the data than the priors. Adeno indicates adenovirus; B. pert, *Bordetella pertussis*; Boca, Human bocavirus; C. pneu, *Chlamydophila pneumoniae;* Cand sp, Candida species; CMV, cytomegalovirus; Entrb, Enterobacteriaceae; Flu, influenza virus A, B and C; H. inf, *Haemophilus influenzae*; HCoV, Coronavirus; Legio, Legionella species; M. cat, *Moraxella catarrhalis*; M. pneu, *Mycoplasma pneumoniae*; Mtb, *Mycobacterium tuberculosis*; NFGNR, nonfermentative gram-negative rods; N. men, *Neisseria meningitidis*; NoS, not otherwise specified (ie, pathogens not tested for); P. jirov, *P. jirovecii*; Para, Parainfluenza virus types 1, 2, 3 and 4; PV/EV, parechovirus/enterovirus; Rhino, human rhinovirus; RSV, respiratory syncytial virus A/B; S. aur, *Staphylococcus aureus*; S. pneu, *Streptococcus pneumoniae*; Salm sp, Salmonella species.

Results of the PIA analysis showed that RSV was the most common pathogen (EF: 26.1%, 95% CrI: 17.0–37.7) among children with CXR-confirmed pneumonia and had double the EF of next most common pathogens, *M. tuberculosis* (12.8%, 95% CrI: 4.3–25.3) and HMPV (12.8%, CrI: 6.1–21.8) (Fig. 2, Supplemental Digital Content 8, http://links.lww.com/INF/D857). In aggregate, viruses accounted for 50.9% (95% CrI: 37.8–64.6) of disease. The top 10 pathogens cumulatively accounted for 84.7% of the etiologic distribution of the cases (95% CrI: 71.6–94.2). Overall, potentially treatable causes of pneumonia accounted for 6 of the top 10 pathogens in Zambia. In descending EF, *M. tuberculosis*, *E. coli* (6.3%, 95% CrI: 0.7–17.7), *H. influenzae* (5.8%, 95% CrI: 0.4–18.5), *Salmonella* (4.6%, 95% CrI: 0.8–12.8), *P. jirovecii* (4.5%, 95% CrI: 0.0–9.6), and *S. aureus* (3.6%, 95% CrI: 0.2–10.1) accounted for 37.6% of pneumonia cases (95% CrI: 24.2–51.2)*. S. pneumoniae* was uncommon, contributing only 1.7% (95% CrI: 0.1–5.6) among (PCV10) vaccine serotypes and <1% for nonvaccine serotypes. Although *Bordetella pertussis* contributed only 2.0% (95% CrI: 0.0–5.3) to the etiologic causes of pneumonia, among the 3 cases positive by NP/OP PCR (Supplemental Digital Content 6, http://links.lww.com/INF/D855), all were young infants (two 1-month and one 3-month old) and 1 died in hospital. When expanding from CXR+ only to all HIV-uninfected children (Supplemental Digital Content 8, http://links.lww.com/INF/D857, Supplemental Digital Content 9, http://links.lww.com/INF/D858), the overall viral to bacterial distribution remained similar with changes observed for some pathogens such as rhinovirus and *H. influenzae* (primarily non-b).

When stratified by age, there were a few notable differences in the distribution of pathogens (Supplemental Digital Content 10, http://links.lww.com/INF/D859, Supplemental Digital Content 11, http://links.lww.com/INF/D860); however, most pathogens had wide and overlapping 95% CrIs as there were only 49 CXR+ children 12–59 months of age. Tuberculosis was more common among infants <1 year (17.1%) than older children (0.9%, mean difference: 16.7; 95% CI: 5.1–32.5), as were *E. coli* (8.2% vs. 1.0%, mean difference: 7.5; 95% CI: −2.6 to 22.3) and *P. jirovecii* (6.0% vs. 0.3%, mean difference: 5.9; 95% CI: 0.0–11.5). Alternatively, Influenza (primarily type A) (1.6% vs. 14.7%, mean difference: −13.9; 95% CI: −29.2 to 0.0) and *S. pneumoniae* (primarily PCV10 vaccine-type) (0.9% vs. 6.3%, mean difference: −5.4; 95% CI: −16.7 to −0.8) were more common in older children. Interestingly, the fraction of pneumonia due to RSV (25.8% vs. 27.0%, mean difference: −2.4; 95% CI: −18.7 to 13.2) and HMPV (12.2% vs. 14.3%, mean difference: −2.7; 95% CI: −17.5 to 12.6) was similar among cases <1 year and ≥1 year of age. Viruses were more common in older children (65.6%; 95% CI: 36.8%–88.7% vs. 45.7%; 95% CI: 31.5%–61.5%).

When stratified by severity, the top 3 pathogens remained the same (Supplemental Digital Content 12, http://links.lww.com/INF/D862, Supplemental Digital Content 13, http://links.lww.com/INF/D863).

### Mortality

While in-hospital follow-up at the Zambia site was available for all children, 1-month follow-up was less complete. Only approximately half of the cases—distributed equally by CXR positivity status—returned for the 1-month visit. We therefore focus here on in-hospital mortality.

There were 82 (16%) deaths among all HIV-uninfected cases, 76 of whom died in hospital. Of the 34 in hospital deaths with an interpretable CXR taken, 23 (67.6%) were CXR positive. Among the 61 children who did not get a CXR, 30 (49%) died in hospital (vs. in-hospital mortality among cases with an interpretable CXR, 9.3%), reflecting their moribund condition upon enrollment (Table 2). In-hospital mortality was higher among cases with very severe pneumonia (case fatality ratio = 28.1%) than for severe (case fatality ratio = 8.8%; *P* < 0.0001). After adjusting for age, the clinical factors significantly associated with fatal pneumonia included malnutrition [weight-for-age Z score < −2, adjusted odds ratios (aOR): 5.0, 95% CI: 2.9–8.3], illness duration at admission >5 days (aOR: 2.2, 95% CI: 1.2–4.1), severe anemia (hemoglobin ≤ 7.5 g/dL, aOR: 2.2, 95% CI: 1.0–4.8), HIV exposure (aOR: 2.1, 95% CI: 1.3–3.6) and leukocytosis (aOR: 1.7, 95% CI: 1.0–2.9) (Table 2). Overall in-hospital mortality among HIV-exposed children was nearly double that in HIV-unexposed cases (21.6% vs. 11.1%, *P* < 0.001). Eight of the 69 fatal cases with results available were blood culture positive; *H. influenzae* and *S. aureus* were most commonly detected (Supplemental Digital Content 5, http://links.lww.com/INF/D854). Pathogens more frequently detected on NP/OP PCR in fatal cases compared with those who survived until discharge included *P. jirovecii* and *H. influenzae* type b (Supplemental Digital Content 6, http://links.lww.com/INF/D855); RSV was significantly less common in fatal cases (7.6%) compared with surviving cases (23.8%) (data not shown).

**TABLE 2.** Clinical and Laboratory Factors Associated With In-hospital Mortality Among All 514 HIV-uninfected Under 5-Year-Old Zambian Children With Severe and Very Severe Pneumonia on Admission

Characteristic	All Cases	Fatal Cases	Nonfatal Cases	aOR*	*P* Value*
Total	514 (100)	76 (100)	438 (100)	—	—
Female	234 (45.5)	41 (53.9)	193 (44.1)	1.5 (0.9–2.4)	0.11
Very severe pneumonia	160 (31.1)	45 (59.2)	115 (26.3)	4.2 (2.5–6.9)	<0.0001
Age <1 year	399 (77.6)	62 (81.6)	337 (76.9)	1.3 (0.7–2.5)	0.3718
Weight for age <−2 SD	160 (31.2)	47 (61.8)	113 (25.9)	4.9 (2.9–8.2)	<0.0001
Weight for height <−2 SD	93 (19.2)	23 (34.9)	70 (16.8)	2.9 (1.6–5.2)	0.0003
Premature†	19 (4.8)	6 (9.8)	13 (3.9)	2.8 (1.0–7.6)	0.048
HIV exposure status					
Exposed/uninfected	134 (26.1)	29 (38.2)	105 (24.0)	2.1 (1.3–3.6)	<0.0001
Unexposed/uninfected	369 (71.8)	41 (53.9)	328 (74.9)	Ref	
Unknown	11 (2.1)	6 (7.9)	5 (1.1)	10.1 (2.9–34.9)	
4 or more people sleeping in the same room	84 (16.4)	22 (29.3)	62 (14.2)	2.5 (1.4–4.5)	0.0012
Duration of illness‡					
≤2 d	181 (35.4)	22 (29.7)	159 (36.4)	Ref	0.012
3–5 d	220 (43.1)	26 (35.1)	194 (44.4)	1.0 (0.5–1.8)	
>5 d	110 (21.5)	26 (35.1)	84 (19.2)	2.2 (1.2–4.1)	
Duration in hospital					
≤2 d	154 (30.0)	57 (75.0)	97 (22.2)	Ref	<0.0001
3–5 d	174 (33.9)	8 (10.5)	166 (38.0)	0.07 (0.03–0.2)	
>5 d	185 (36.1)	11 (14.5)	174 (39.8)	0.10 (0.05–0.2)	
CXR positive§					
Consolidation or other infiltrate	208 (56.7)	23 (67.6)	185 (55.6)	1.7 (0.8–3.5)	0.19
Normal	159 (43.3)	11 (32.4)	148 (44.4)	Ref	
Runny nose	180 (35.0)	11 (14.5)	169 (38.6)	0.2 (0.1–0.5)	<0.0001
Hypoxia¶	185 (36.1)	46 (60.5)	139 (31.8)	3.3 (2.0–5.4)	<0.0001
Lethargy	64 (12.5)	32 (42.1)	32 (7.3)	10.2 (5.6–18.5)	<0.0001
Deep breathing	36 (7.0)	12 (15.8)	24 (5.5)	3.4 (1.6–7.2)	0.0013
Observed cough	349 (67.9)	37 (48.7)	312 (71.2)	0.4 (0.2–0.6)	0.0001
Observed grunting	137 (26.7)	36 (47.4)	101 (23.1)	3.0 (1.8–4.9)	<0.0001
Severe anemia‖	41 (8.2)	10 (14.3)	31 (7.2)	2.2 (1.0–4.8)	0.041
Leukocytosis**	213 (42.7)	38 (53.5)	175 (40.9)	1.7 (1.0–2.9)	0.032

*aOR and *P*-values adjusted for age in months.

†Prematurity defined as <37 weeks gestational age or maternal report of premature.

‡Duration of illness defined as duration (in days) of cough, wheeze, fever or difficulty breathing, whichever is longest.

§Restricted to those with an interpretable CXR (N = 34 for fatal cases and N = 333 for nonfatal cases). CXR obtained at admission.

¶Hypoxemia defined as oxygen saturation <90% or on supplemental oxygen if a room air oxygen saturation reading was not available.

‖Severe anemia defined as hemoglobin ≤7.5 g/dL.

**Leukocytosis count defined as >15 × 10^9^ cells/L for children 1–11 months and >13 × 10^9^ cells/L for children 12–59 months.

**FIGURE 3.**
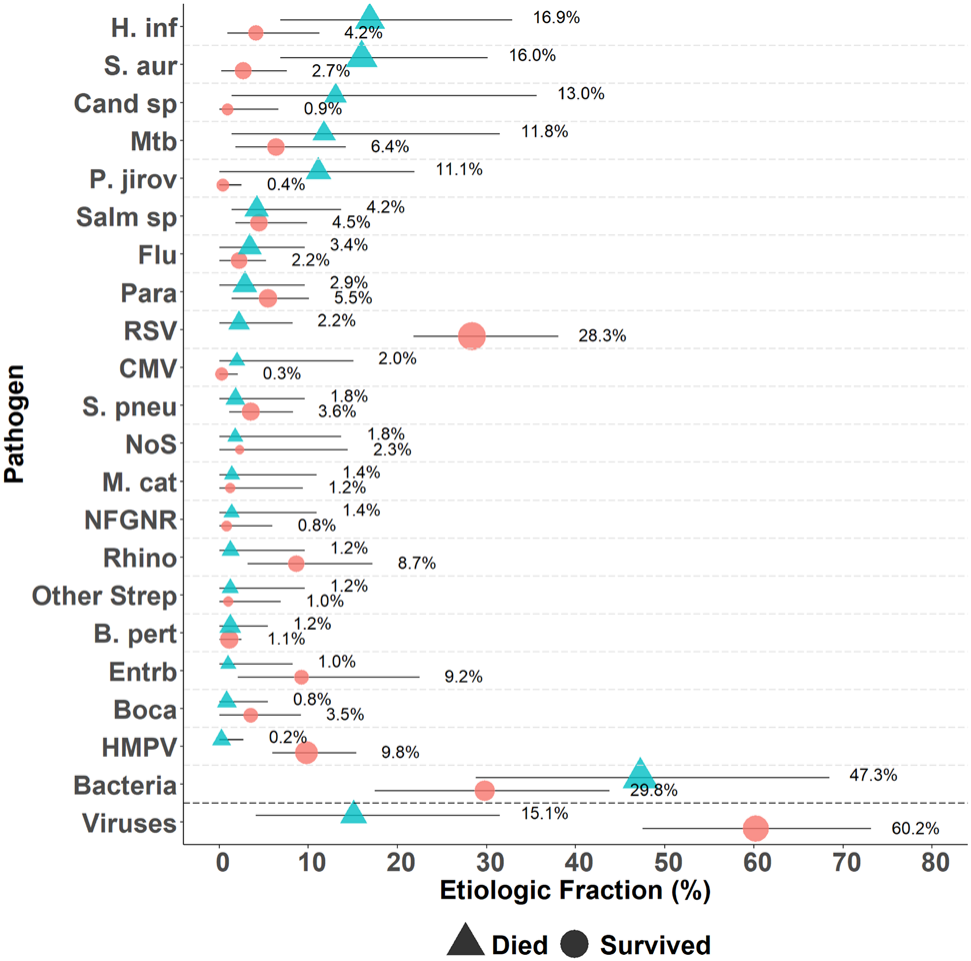
Etiologic fraction of pneumonia among 514 HIV-uninfected cases of pneumonia regardless of finding on radiograph, stratified by in-hospital death or survival. Pathogens with low etiologic fraction from the CXR+/HIV− analysis are excluded from this figure (*Neisseria meningitidis*, Adenovirus, parechovirus/enterovirus, human Coronavirus, *Mycoplasma pneumoniae*, Legionella, and *Chlamydophila pneumoniae*). Other Strep includes *Streptococcus pyogenes* and *Enterococcus faecium*. NFGNR includes Acinetobacter species and Pseudomonas species. Enterobacteriaceae includes *E. coli*, Enterobacter species, and Klebsiella species, excluding mixed gram-negative rods. Bacterial summary excludes Mtb. Analysis adjusted for age and vital status. Pathogens estimated at the subspecies level but grouped to the species level for display (Parainfluenza virus type 1, 2, 3 and 4; *S. pneumoniae* PCV10 and *S. pneumoniae* non-PCV10 types; *H. influenzae* type b and *H. influenzae* non-b; influenza A, B, and C). Description of symbols: Line represents the 95% credible interval. The size of the symbol is scaled based on the ratio of the estimated etiologic fraction to its standard error. Of 2 identical etiologic fraction estimates, the estimate associated with a larger symbol is more informed by the data than the priors. B. pert indicates *Bordetella pertussis*; Boca, Human bocavirus; Cand sp, Candida species; CMV, cytomegalovirus; Entrb, Enterobacteriaceae; Flu, influenza virus A, B and C; H. inf, *Haemophilus influenzae*; M. cat, *Moraxella catarrhalis*; M. pneu, *Mycoplasma pneumoniae*; Mtb, *Mycobacterium tuberculosis*; NFGNR, nonfermentative gram-negative rods; NoS, not otherwise specified (ie, pathogens not tested for); P. jirov, *P. jirovecii*; Para, Parainfluenza virus types 1, 2, 3 and 4; Rhino, human rhinovirus; RSV, respiratory syncytial virus A/B; S. aur, *Staphylococcus aureus*; S. pneu, *Streptococcus pneumoniae*; Salm sp, Salmonella species.

Given the large number of fatal cases missing a CXR, we estimated the etiology among all HIV-uninfected cases, stratified by mortality status, to include 6 of 8 fatal cases with a positive blood culture who were missing CXR (Supplemental Digital Content 5, http://links.lww.com/INF/D854, Fig. 3). The cause of pneumonia among fatal cases was predominantly non-*M. tuberculosis* bacterial, comprising nearly half (47.3%, 95% CI: 28.8–68.5) of the cases who died in hospital compared with 29.8% (95% CI: 17.4–43.8) for cases who were discharged. *H. influenzae* type b (6.2% died vs. 1.1% survived, mean difference: 5.2; 95% CI: 0.0–13.0), *S. aureus* (16.0% v. 2.7%, mean difference: 13.1; 95% CI: 2.7–27.6), *P. jirovecii* (11.1% vs. 0.4%, mean difference: 10.7; 95% CI: 0.0–21.9) and fungi (13.0% vs. 0.9%, mean difference: 12.1; 95% CI: 0.0–34.2) were associated with death, while RSV (2.2% vs. 28.3%, mean difference: 26.0; 95% CI: −36.3 to −17.6), rhinovirus (1.2% vs. 8.7%, mean difference: −7.4; 95% CI: −16.4 to 1.6) and HMPV (0.2% vs. 9.8%, mean difference: −9.5; 95% CI: −15.1 to −5.5) were associated with survival to discharge. Although the fraction of pneumonia due to *M. tuberculosis* did not differ by mortality status (11.8% vs. 6.4%, mean difference: 5.4; 95% CI: −8.0 to 25.8), it was a common cause of pneumonia among children who died.

## DISCUSSION

The clinical and etiology findings described here reflect the presentation of community-acquired childhood pneumonia among HIV-uninfected children in the setting of a typical sub-Saharan African city, Lusaka, Zambia—an impoverished, densely populated, urban population with high HIV and *M. tuberculosis* prevalences and limited access to high-quality health care. Our results should be contrasted with the findings from the 4 other sub-Saharan African PERCH sites with higher-quality health care (South Africa), little HIV (Kenya/Mali/Gambia) and a rural population (Kenya/Gambia).

The main findings at the Zambia PERCH site include (1) the commonest pathogens contributing to severe and very severe pneumonia are similar to the other PERCH sites from Africa and Asia; (2) RSV is the dominant cause of pneumonia, across age and in both WHO severity strata; (3) CXR+ pneumonia was caused by treatable organisms in a large portion (37.6%) of the cases; (4) pediatric *M. tuberculosis* is a significant cause of pneumonia for hospitalized children; and (5) children with severe and very severe pneumonia have a very poor prognosis in Zambia.

Although PERCH was designed to assess the presentation of childhood pneumonia in globally diverse geographic settings, it is noteworthy that we observed relatively little variation in the most prevalent pneumonia pathogens. In Zambia, we found that a viral etiology was a somewhat less common cause of pneumonia (EF: 50.9%) than at all the PERCH sites (EF: 61.4%) reported in the all-sites analysis.^22^ Similar to the other PERCH sites, the 10 most frequently identified pneumonia pathogens in Zambia accounted for over 80% of causes for pneumonia. The remaining 22 pathogens assessed in this study accounted for a small portion of the remaining cases of pneumonia.

RSV was the dominant cause of pneumonia in Zambia (EF: 26.1%) as it was in the other PERCH sites and exhibited a clearly seasonal pattern. This is consistent with findings from numerous sources.^25–27^ However, it is notable that RSV contributes relatively little to the pneumonia mortality. Nevertheless, these findings underscore the importance of developing prevention and treatment strategies for RSV in Zambia and support the importance of developing an effective vaccine. Current treatment for severe RSV disease is solely focused on supportive care, including oxygen, which can be in short supply in Zambia.

Vaccine-preventable diseases (*Haemophilus influenzae* type b, vaccine-type *S. pneumoniae*, pertussis) were relatively rare among the PERCH cases (EF: 6.8, 95% CI: 0.02–14.4). Disease due to *B. pertussis* was infrequent (EF: 2.0%), presumably due in part to very high DTP vaccination rates (≈80%), but was more common in infants, an age group of higher pertussis mortality. Despite only a handful of children being fully immunized to PCV10 in Zambia, disease due to PCV10 serotypes of *S. pneumoniae* was infrequent (EF: 1.7%).

Potentially treatable causes for pneumonia comprised a large proportion of cases in Zambia. Six of the top 10 pathogens at our site (descending order—*M. tuberculosis*, *E. coli*, *H. influenzae* type b, *Salmonella* species, *P. jirovecii* and *S. aureus*) accounted for almost 40% of disease. Empiric antibiotic treatment, for commonly presenting bacterial pathogens, needs to be updated accordingly and guided by local antibiotic resistance patterns. Additional trimethoprim-sulfamethoxazole coverage for *P. jirovecii*, particularly for under-1 children exposed to HIV, should also be emphasized in Zambia. Further efforts for detecting *M. tuberculosis* in all children presenting with pneumonia in Zambia should be considered.

Overall in-hospital mortality among HIV-exposed-uninfected children was nearly double that in HIV-unexposed-uninfected cases (22% vs. 11%, *P* < 0.001). This may be due to the lower viral etiology (EF: 30.3% HIV exposed vs. 53.2% HIV unexposed), and higher bacterial (*S. aureus*, *Salmonella* species and *E. coli*) causes, along with *P. jirovecii*, among the HIV-exposed children.^7^ In a nested substudy, we have previously shown that standardized empiric therapy for these HIV-exposed children improves outcome.^28^ *P. jirovecii* was somewhat common (EF: 4.5% in CXR+ cases) among this HIV-uninfected cohort reported here and was associated with high in-hospital mortality. However, most of the *P. jirovecii* was found in the HIV-exposed children (10.9% vs. 0.7% HIV unexposed).^7^

*M. tuberculosis* was one of the most common pathogens found at our site and was estimated to cause 12.8% of pneumonia CXR+/HIV-uninfected cases. *M. tuberculosis* as a common cause of pneumonia is consistent with others^29^ who found a 7.5% prevalence of culture-confirmed *M. tuberculosis* among cases of severe pneumonia. This is also consistent with a recent autopsy study from UTH which found pulmonary tuberculosis in 8% of in-hospital deaths.^30^ Tuberculosis is a potentially preventable and treatable disease in children, despite the difficulties in diagnosis, and ongoing efforts for improving diagnosis (use of induced sputum, multiple sputum samples; GeneXpert, Cepheid, Sunnyvale, CA), prevention (isoniazid for exposed infants, for example) and care (long-term appropriate antituberculosis regimens) should be made to improve child survival.

Our in-hospital case fatality ratio (CFR) (14.8%) is the highest among the 9 PERCH sites (11.1%) but close to the Mali site (10.4%). The increased CFR is partly explained by the high prevalence of well-established risk factors for poor outcomes for childhood pneumonia: malnutrition, very severe disease as defined by presence of danger signs, and hypoxia on presentation. Delayed access to care, evidenced by the longer duration of illness among the fatal cases, was also a factor. Other probable contributors to the higher mortality were the limited availability of mechanical ventilation, preponderance of bacterial etiologies and lack of standardized care among the pediatric hospital staff.^28^ Nonetheless, with pulse oximetry more widely available, it is important to note that easily assessable information on hypoxia and WHO danger signs on admission are early indicators of children with pneumonia who are at high risk of death, and thus easy areas of focus when trying to improve outcomes.

There were limitations to the PERCH study, such as reliance on samples obtained from outside the lung and challenges distinguishing NP common carriage from infection, as discussed in the all-site paper.^22^ Additionally, the PIA model assumes each case’s pneumonia episode is caused by a single pathogen and it does not attempt to identify or quantify pathogen combinations.^22^ The Zambia site had several other limitations. First, less than half of the cases (CXR+ and CXR normal) were seen at 1 month posthospitalization, which likely underestimated the mortality. Second, our rate of antibiotic pretreatment was high, owing to the near universal practice of giving 1 dose of antibiotic before referral to UTH, which likely diminished the isolation of bacterial pathogens from blood culture. Positive blood culture results are a key input into the PIA, and despite lowering the blood culture sensitivity priors in the PIA for those on antibiotics, the analysis may have underestimated bacterial pathogens. Third, 12% of cases did not have a CXR performed. Timely radiography was a challenge because radiographers were sometimes unavailable due to staffing issues or were located across campus, a particular issue for the sickest children who died shortly after enrolment. Fourth, we did not perform any autopsies on fatal cases at our site. Nonetheless, the distribution of pathogens found in pneumonia deaths in an in-hospital autopsy study conducted during the same period at UTH were similar,^30^ where a large proportion of children had histopathologic evidence of bronchopneumonia (50%), mycobacteria (8%) and *P. jirovecii* (5%). Children enrolled in the autopsy study were slightly older (median age: 19 months) than PERCH cases (median age: 5 months) but had similar comorbidities, including high severe malnutrition and HIV prevalence. Fifth, to improve the specificity of WHO-defined pneumonia that comprised our inclusion criteria, the PERCH study chose to define cases based on a positive CXR for the primary analysis. We may have excluded some cases of true pneumonia among those subjects who died before obtaining a CXR. Finally, we may have omitted cases of true pneumonia that developed a positive CXR after admission or omitted some cases of pneumonia (*P. jirovecii*) which may not have had any CXR findings.

The results presented here provide a clinical and microbiologic view of HIV-uninfected children with community-acquired pneumonia in an urban setting in a typical sub-Saharan African city. We have purposely omitted the HIV-infected cases (described in detail elsewhere) to provide a sample of children more comparable with the other PERCH sites (except South Africa). In aggregate, our results show a similar etiologic spectrum of pneumonia to the other PERCH sites, albeit a higher mortality. It remains to be determined how much of this increased mortality is due to drug resistance, more virulent organism or health system effects.

## ACKNOWLEDGMENTS

We offer sincere thanks to the children and families who participated in this study. We acknowledge the work of all Pneumonia Etiology Research for Child Health (PERCH) Contributors who were involved in data collection at the local sites and central laboratories, members of the PERCH Chest Radiograph Reading Panel, and Shalika Jayawardena and Rose Watt from Canterbury Health Laboratories. We also acknowledge the substantial contributions of the other members of the PERCH Study Group not listed as coauthors: Johns Hopkins Bloomberg School of Public Health, Baltimore, MD: Orin S. Levine (Former PI, current affiliation Bill & Melinda Gates Foundation, Seattle, WA), Andrea N. DeLuca, Amanda J. Driscoll, Nicholas Fancourt, Wei Fu, E. Wangeci Kagucia, Ruth A. Karron, Mengying Li, Daniel E. Park, Qiyuan Shi, Zhenke Wu, Scott L. Zeger; The Emmes Corporation, Rockville, MD: Nora L. Watson; Nuffield Department of Clinical Medicine, University of Oxford, United Kingdom: Jane Crawley; Medical Research Council, Basse, The Gambia: Stephen R. C. Howie (site PI); KEMRI-Wellcome Trust Research Programme, Kilifi, Kenya: J. Anthony G. Scott (site PI and PERCH co-PI, joint affiliation with London School of Hygiene and Tropical Medicine, London, United Kingdom); Division of Infectious Disease and Tropical Pediatrics, Department of Pediatrics, Center for Vaccine Development and Global Health, University of Maryland School of Medicine, Baltimore, MD and Centre pour le Développement des Vaccins (CVD-Mali), Bamako, Mali: Karen L. Kotloff (site PI); Medical Research Council: Respiratory and Meningeal Pathogens Research Unit and Department of Science and Technology/National Research Foundation: Vaccine Preventable Diseases, University of the Witwatersrand, Johannesburg, South Africa: Shabir A. Madhi (site PI); International Centre for Diarrhoeal Disease Research, Bangladesh (icddr,b): W. Abdullah Brooks (site PI); Thailand Ministry of Public Health—US CDC Collaboration, Nonthaburi, Thailand: Henry C. Baggett (site PI), Susan A. Maloney (former site PI); and Canterbury Health Laboratories, Christchurch, New Zealand: Trevor P. Anderson, Joanne Mitchell.

## Supplementary Material


