# Supplementary material for: The Etiology of Pneumonia in Zambian Children: Findings From the Pneumonia Etiology Research for Child Health (PERCH) Study
Source: Pediatr Infect Dis J. 2021 Aug 25;40(9):S40–9. doi: 10.1097/INF.0000000000002652 (PMC8448410; doi:10.1097/INF.0000000000002652)
Supplement: Supplementary file 1 [file inf-40-s40-s001.docx]

**Supplemental Digital Content 1. Supplemental study methods and site- and country-level characteristics**

**Methods**

*Location*

The PERCH Zambia site enrolled children in Lusaka, Zambia’s capital city. Cases were children hospitalized at the University Teaching Hospital (UTH). The UTH Pediatric Ward has 425 inpatient beds, including dedicated Malnutrition and Intensive Care Units.

*Study participants*

Children 1-59 months of age presenting to University Teaching Hospital (UTH) between November 2011 and October 2013 with severe or very severe pneumonia were eligible to be cases. Severe pneumonia was defined as presence of cough or difficulty breathing plus lower chest wall indrawing. Very severe pneumonia was defined as cough or difficulty breathing and one or more danger signs (central cyanosis, difficulty breastfeeding/drinking, vomiting everything, convulsions, lethargy, reduced consciousness, or head nodding). Cases were excluded if they had been hospitalized within the previous 14 days, were a PERCH study participant within 30 days, or had lower chest wall in-drawing (LCWI) resolve following bronchodilator challenge for children with auscultatory wheeze and lower chest wall indrawing in the absence of danger signs.

Controls were randomly selected from the community. A target number of controls were enrolled each month, aiming for a 1:1 ratio and age-matched to cases using four strata: one to 5 months, 6 to 11 months, 12 to 23 months, and 24 to 59 months. Controls were excluded if they had been a PERCH case within the previous 30 days, had been hospitalized within the previous 14 days, or had case defining symptoms. They were not excluded if they had respiratory symptoms.

*Specimen Collection and Laboratory Methods*

Blood specimens were collected and tested for complete blood count, HIV, malaria, C-Reactive Protein (CRP), blood culture (cases only), antibiotic activity, and whole blood lytA PCR for Streptococcus pneumoniae. Naso- and Oro-pharyngeal swabs (NP/OP) were collected in both cases and controls for routine culture and quantitative PCR (FTD Resp-33 Kit, Fast-track Diagnostics, Sliema, Malta). In cases only, induced sputa were collected and tested for Mycobacteria tuberculosis (MTB), and pleural fluid samples were obtained, if clinically indicated, and tested for culture and quantitative PCR. All children were tested for malaria using a bedside rapid diagnostic test (Paracheck® PF-Rapid Test, Orchid Biomedical Systems). HIV exposure status of the child (and mother) was obtained from the child’s health card, if available. If card was not available, the mothers were asked about their HIV status and offered HIV serology testing. All exposed children had HIV testing performed by DNA PCR or serology, depending on age (+/- 18 months) and per Zambian National HIV Guidelines for children.

**Table. PERCH Zambia Site and Country Characteristics**

| **Site-level characteristics** | **Site** | Lusaka |
| --- | --- | --- |
|  | **Country** | Zambia |
|  | **Urban/Rural** | Urban |
|  | **Population, Thousands^a^** | 1700 |
|  | **PERCH Enrollment Dates** | October 2011 - October 2013 |
| **Country-level characteristics** | **2012 Population, Thousands^b^** | 14,075 |
|  | **2012 GNI Per Capita, US$^b^** | 1,350 |
|  | **2012 U5 Mortality Rate^b^** | 89 |
|  | **2012 Infant Mortality Rate^b^** | 56 |
|  | **2012 HIV Infection Prevalence Among Women 15–24 Years Old^c^** | 4.6% ^d^ |
|  | **Malaria Prevalence^d^** | 4.8% |
|  | **Hib Vaccine Introduction Date^e^** | Feb-04 |
|  | **2012 Hib 3 dose coverage^f^** | 78% |
|  | **PCV Introduction Date^e^** | May 2013^g^ |

Abbreviations: GNI, gross national income; Hib, *Haemophilus influenzae* type b; HIV, human immunodeficiency virus; PCV, pneumococcal conjugate vaccine; U5, under 5.

^a.^ Central statistics office. 2010 Census of population and housing. Natl Anal Rep. 2012:1-117. <http://www.zamstats.gov.zm/census/cen.html>.

^b^ Per 1,000 live births. Applies to country. Source: United Nations Children’s Fund, State of the World’s Children 2014 (<https://www.unicef.org/sowc2014/numbers/>).

^c^ HIV infection prevalence amongst women 15-49 was 15.1% in Zambia (2013-2014).

^d^ Based on rapid diagnostic test. Lusaka Province, children under 5 years of age. Source: 2012 Malaria Indicator Survey.

^e^ Applies to country. Source: International Vaccine Access Center (IVAC), Johns Hopkins Bloomberg School of Public Health. Vaccine Information Management System (VIMS) Global Vaccine Introduction Report, [December 2015]. http://www.jhsph.edu/research/centers-and-institutes/ivac/vims/. Accessed [10 March 2017].

^f^ Applies to country. Source: WHO vaccine-preventable diseases: monitoring system (<http://apps.who.int/immunization_monitoring/globalsummary/estimates?c=ZMB>).

^g^ Introduced in Lusaka, Zambia in July 2013 (3 months prior to end of enrollment at the site).
