# Supplementary material for: The Etiology of Pneumonia in Zambian Children: Findings From the Pneumonia Etiology Research for Child Health (PERCH) Study
Source: Pediatr Infect Dis J. 2021 Aug 25;40(9):S40–9. doi: 10.1097/INF.0000000000002652 (PMC8448410; doi:10.1097/INF.0000000000002652)
Supplement: Supplementary file 3 [file inf-40-s40-s003.docx]

**Supplemental Digital Content 3, Figure. Seasonality of enrollment and RSV NP/OP PCR positivity among** **HIV-uninfected cases with severe and very severe pneumonia and controls**

Abbreviations: NP/OP, nasopharyngeal/oropharyngeal; RSV, Respiratory syncytial virus A/B.
