# Supplementary material for: The Etiology of Pneumonia in Zambian Children: Findings From the Pneumonia Etiology Research for Child Health (PERCH) Study
Source: Pediatr Infect Dis J. 2021 Aug 25;40(9):S40–9. doi: 10.1097/INF.0000000000002652 (PMC8448410; doi:10.1097/INF.0000000000002652)
Supplement: Supplementary file 4 [file inf-40-s40-s004.docx]

**Supplemental Digital Content 4, Table. Demographic and Clinical Characteristics of HIV-Uninfected cases with severe and very severe pneumonia, Stratified by CXR finding and Severity**

|  | **All Cases** | **By CXR Finding** | | **All Cases by Severity** | |
| --- | --- | --- | --- | --- | --- |
|  |  | **CXR+ Cases** | **CXR-Normal Cases** | **Severe Cases** | **Very Severe Cases** |
| **All** | 514 | 208 | 159 | 354 | 160 |
| **Age** |  |  |  |  |  |
| **1 - 5 m** | 276 (53.7) | 110 (52.9) | 88 (55.3) | 190 (53.7) | 86 (53.8) |
| **6 - 11 m** | 123 (23.9) | 49 (23.6) | 34 (21.4) | 91 (25.7) | 32 (20) |
| **12 - 23 m** | 76 (14.8) | 37 (17.8) | 22 (13.8) | 47 (13.3) | 29 (18.1) |
| **24 - 59 m** | 39 (7.6) | 12 (5.8) | 15 (9.4) | 26 (7.3) | 13 (8.1) |
| **Female** | 234 (45.5) | 91 (43.8) | 79 (49.7) | 160 (45.2) | 74 (46.3) |
| **Very severe pneumonia** | 160 (31.1) | 70 (33.7) | 44 (27.7) | 0 (0.0) | 160 (100) |
| **CXR available** | 453 (88.1) | 208 (100) | 159 (100) | 316 (89.3) | 137 (85.6) |
| **CXR result** |  |  |  |  |  |
| **Any consolidation** | 135 (29.8) | 135 (64.9) | 0 (0.0) | 85 (26.9) | 50 (36.5) |
| **Other infiltrate only** | 73 (16.1) | 73 (35.1) | 0 (0.0) | 53 (16.8) | 20 (14.6) |
| **Normal** | 159 (35.1) | 0 (0.0) | 159 (100) | 115 (36.4) | 44 (32.1) |
| **Uninterpretable** | 86 (19) | 0 (0.0) | 0 (0.0) | 63 (19.9) | 23 (16.8) |
| **Season of enrolment** |  |  |  |  |  |
| **Dry (June-October)** | 201 (39.1) | 92 (44.2) | 64 (40.3) | 126 (35.6) | 75 (46.9) |
| **Rainy (November-May)** | 313 (60.9) | 116 (55.8) | 95 (59.7) | 228 (64.4) | 85 (53.1) |
| **HIV exposure status** |  |  |  |  |  |
| **Exposed/uninfected** | 134 (26.1) | 55 (26.4) | 45 (28.3) | 99 (28) | 35 (21.9) |
| **Unexposed/uninfected** | 369 (71.8) | 151 (72.6) | 113 (71.1) | 248 (70.1) | 121 (75.6) |
| **Unknown** | 11 (2.1) | 2 (1.0) | 1 (0.6) | 7 (2.0) | 4 (2.5) |
| **Weight-for-age (WHO)** |  |  |  |  |  |
| **>-2 z scores** | 353 (68.8) | 142 (68.3) | 118 (74.7) | 245 (69.4) | 108 (67.5) |
| **< -2 scores** | 160 (31.2) | 66 (31.7) | 40 (25.3) | 108 (30.6) | 52 (32.5) |
| **Median duration of illness^a^ in days (IQR)** | 3 (2, 5) | 3 (2, 6) | 3 (2, 4) | 3 (2, 5) | 3 (2, 5) |
| **Duration of illness at enrolment^a^** |  |  |  |  |  |
| **0 - 2 days** | 181 (35.4) | 64 (30.8) | 64 (40.8) | 130 (36.8) | 51 (32.3) |
| **3 - 5 days** | 220 (43.1) | 91 (43.8) | 68 (43.3) | 151 (42.8) | 69 (43.7) |
| **>5 days** | 110 (21.5) | 53 (25.5) | 25 (15.9) | 72 (20.4) | 38 (24.1) |
| **Median duration of hospitalization in days (IQR)** | 4 (2, 7) | 5 (3, 8) | 4 (3, 6) | 4 (2, 7) | 5 (2, 8) |
| **Duration of hospitalization** |  |  |  |  |  |
| **0 - 2 days** | 141 (27.5) | 36 (17.4) | 36 (22.6) | 93 (26.3) | 48 (30.2) |
| **3 - 5 days** | 176 (34.3) | 73 (35.3) | 66 (41.5) | 135 (38.1) | 41 (25.8) |
| **>5 days** | 196 (38.2) | 98 (47.3) | 57 (35.8) | 126 (35.6) | 70 (44.0) |
| **Hypoxemia^b^** | 185 (36.1) | 97 (46.6) | 35 (22.2) | 105 (29.7) | 80 (50.0) |
| **Tachypnea^c^** | 443 (87.0) | 188 (90.8) | 131 (84.5) | 312 (88.9) | 131 (82.9) |
| **Tachycardia^d^** | 328 (64.6) | 135 (65.2) | 101 (64.7) | 225 (64.5) | 103 (64.8) |
| **Head nodding** | 69 (13.4) | 31 (14.9) | 21 (13.2) | 0 (0.0) | 69 (43.1) |
| **Central cyanosis** | 18 (3.5) | 8 (3.8) | 4 (2.5) | 0 (0.0) | 18 (11.3) |
| **Convulsions** | 25 (4.9) | 9 (4.3) | 8 (5.0) | 0 (0.0) | 25 (15.6) |
| **Lethargy** | 64 (12.5) | 28 (13.5) | 15 (9.4) | 0 (0.0) | 64 (40) |
| **Unable feed** | 44 (8.6) | 16 (7.7) | 9 (5.7) | 0 (0.0) | 44 (27.5) |
| **Vomiting** | 4 (0.8) | 0 (0.0) | 3 (1.9) | 0 (0.0) | 4 (2.5) |
| **Crackles** | 294 (57.2) | 117 (56.3) | 93 (58.5) | 193 (54.5) | 101 (63.1) |
| **Wheeze on auscultation** | 63 (12.3) | 22 (10.6) | 20 (12.6) | 34 (9.6) | 29 (18.1) |
| **Grunting** | 137 (26.7) | 56 (26.9) | 45 (28.3) | 82 (23.2) | 55 (34.4) |
| **Nasal flaring** | 319 (62.1) | 127 (61.1) | 97 (61.0) | 213 (60.2) | 106 (66.3) |
| **Elevated temperature (> 38 C)** | 267 (52.1) | 122 (58.9) | 74 (46.5) | 177 (50.1) | 90 (56.6) |
| **Leukocytosis^e^** | 213 (42.7) | 97 (47.8) | 53 (34.4) | 140 (40.7) | 73 (47.1) |
| **CRP > 40 mg/L** | 165 (35.6) | 91 (48.4) | 35 (24.3) | 119 (37.2) | 46 (32.2) |
| **Severe anemia^f^** | 41 (8.2) | 18 (8.9) | 9 (5.8) | 22 (6.4) | 19 (12.3) |
| **Antibiotic pretreatment prior to specimen collection^g^** | 462 (91.5) | 189 (92.2) | 143 (91.1) | 324 (92.8) | 138 (88.5) |
| **Serum antibiotic activity** | 141 (28.8) | 50 (25.3) | 39 (25.5) | 100 (29.3) | 41 (27.5) |
| **Died in hospital or within 30 days of admission** | 82 (16.0) | 25 (12.05) | 14 (8.8) | 36 (10.2) | 46 (28.8) |
| **Died in-hospital** | 76 (14.8) | 23 (11.1) | 11 (6.9) | 31 (8.8) | 45 (28.1) |
| **Died within 24 hours of admission** | 37 (7.2) | 6 (2.9) | 1 (0.6) | 16 (4.5) | 21 (13.1) |
| **Died post-discharge, within 30 days of admission^h^** | 6 (2.7) | 2 (1.9) | 3 (4.2) | 5 (2.6) | 1 (1.5) |
| **Died within 7 days of discharge^h^** | 2 (0.9) | 1 (1.0) | 0 (0.0) | 1 (0.6) | 1 (1.5) |
| **Missing 30-day vital status** | 216 (42.0) | 82 (39.4) | 77 (48.4) | 166 (46.9) | 50 (31.3) |

Abbreviations: CRP, C-reactive protein; m (months); IQR, interquartile range; CXR, chest radiograph; HIV, human immunodeficiency virus.

a. Duration of illness defined as duration (in days) of cough, wheeze, fever, or difficulty breathing, whichever is longest.

b. Hypoxaemia defined as oxygen saturation <90% or on supplemental oxygen if a room air oxygen saturation reading was not available.

c. Tachypnoea defined as ≥60 breaths/min (<2 months), ≥50 breaths/min (2-11 months), and ≥40 breaths/min (12-59 months).

d. Tachycardia defined as > 160 bpm (0-11 months), > 150 bpm (12-35 months), >140 bpm (36-59 months).

e. Leukocytosis defined as >15 x 10^9^cells/L for children 1-11 months and >13 x 10^9^ cells/L for children 12-59 months.

f. Defined as Hemoglobin 0-7.5 g/dL.

g. Defined as serum bioassay positive, antibiotics administered at the referral facility, or antibiotic administration prior to whole-blood specimen collection at the study facility.

h. Restricted to children discharged alive who had vital status data obtained >21days following admission.
