# Supplementary material for: The Etiology of Pneumonia in Zambian Children: Findings From the Pneumonia Etiology Research for Child Health (PERCH) Study
Source: Pediatr Infect Dis J. 2021 Aug 25;40(9):S40–9. doi: 10.1097/INF.0000000000002652 (PMC8448410; doi:10.1097/INF.0000000000002652)
Supplement: Supplementary file 5 [file inf-40-s40-s005.docx]

**Supplemental Digital Content 5, Table. Organisms detected in case-only blood and sputum specimens from HIV-uninfected cases with severe and very severe pneumonia**

| **Blood Culture** |  |  |  |  |
| --- | --- | --- | --- | --- |
| **ORGANISM** | **All Cases N=505** | **Died in Hospital^b^ N=69** | **CXR+ Cases N=205** |  |
| **Any^a^** | 24 (4.8) | 8 (11.6) | 8 (3.9) |  |
| ***S. pneumoniae*** | 3 (0.6) | 0 (0.0) | 1 (0.5) |  |
| ***S. pneumoniae* VT (PCV10)** | 3 (0.6) | 0 (0.0) | 1 (0.5) |  |
| ***S. pneumoniae* non-VT (PCV10)** | 0 (0.0) | 0 (0.0) | 0 (0.0) |  |
| ***H. influenzae*** | 5 (1.0) | 3 (4.3) | 1 (0.5) |  |
| ***H. influenzae* type b** | 2 (0.4) | 1 (1.4) | 1 (0.5) |  |
| ***H. influenzae* non-type b** | 3 (0.6) | 2 (2.9) | 0 (0.0) |  |
| ***S. aureus*** | 4 (0.8) | 3 (4.3) | 1 (0.5) |  |
| **Salmonella species^c^** | 7 (1.4) | 1 (1.4) | 3 (1.5) |  |
| ***E. coli*** | 4 (0.8) | 0 (0.0) | 2 (1.0) |  |
| **Candida species** | 1 (0.2) | 1 (1.4) | 0 (0.0) |  |
| **Induced sputum culture** | |  |  |  |
| **ORGANISM** | | **All Cases N=422** | **Died in Hospital N=27** | **CXR+ Cases N=182** |
| ***Mycobacterium tuberculosis*** | | 5 (1.2) | 1 (3.7) | 5 (2.7) |

Abbreviations: PCV, pneumococcal conjugate vaccine.

a. Excluding contaminants.

b. Of the 76 children who died in hospital, 69 (90.8%) had a blood culture specimen obtained. Of those, 26 (37.7%) were missing a chest radiograph.

c. Includes *S. typhi*, and Other Salmonella species
