# Supplementary material for: The Etiology of Pneumonia in Zambian Children: Findings From the Pneumonia Etiology Research for Child Health (PERCH) Study
Source: Pediatr Infect Dis J. 2021 Aug 25;40(9):S40–9. doi: 10.1097/INF.0000000000002652 (PMC8448410; doi:10.1097/INF.0000000000002652)
Supplement: Supplementary file 6 [file inf-40-s40-s006.docx]

**Supplemental Digital Content 6, Table: Detection of Organisms in NP/OP and Whole Blood Specimens from HIV-uninfected Cases with severe and very severe pneumonia and Controls, prevalence and odds ratios**

|  | | | | | | | | **Odds Ratio (95% CI)** | | |
| --- | --- | --- | --- | --- | --- | --- | --- | --- | --- | --- |
|  | **All Cases** | | **CXR+ Cases** | | **All Controls** | | **All Cases dying in hospital** | **All Cases vs. All Controls** | | **CXR+ Cases vs. All Controls** |
| **Pathogen** | | | | | | | | | | |
| **Any Pathogen** | | 462 (97.7) | 188 (97.4) | | | 511 (96.4) | 65 (98.5) | 1.16 (0.51, 2.64) | 0.85 (0.28, 2.56) | |
| **Any pathogen, above threshold** | | 445 (94.1) | 181 (93.8) | | | 486 (91.7) | 61 (92.4) | 1.07 (0.57, 2.02) | 0.81 (0.34, 1.96) | |
| **Bacteria** | | | | | | | | | | |
| **Any bacteria** | 437 (92.4) | | 180 (93.3) | | 498 (94.0) | | 59 (89.4) | 0.68 (0.37, 1.27) | | 0.67 (0.28, 1.60) |
| **Any bacteria, with thresholds applied for *S. pneumoniae, H. influenzae*** | 375 (79.3) | | 155 (80.3) | | 453 (85.5) | | 50 (75.8) | 0.40 (0.19, 0.83) | | 0.46 (0.17, 1.25) |
| ***S. pneumoniae*** |  | |  | |  | |  |  | |  |
| **Any positivity** | 365 (77.2) | | 150 (77.7) | | 429 (80.9) | | 52 (78.8) | 0.80 (0.55, 1.16) | | 0.80 (0.47, 1.36) |
| **>6.9 log_10_ copies/ml** | 34 (7.2) | | 15 (7.8) | | 29 (5.5) | | 4 (6.1) | 1.40 (0.76, 2.56) | | 1.42 (0.66, 3.08) |
| **Among those with >6.9 log_10_ copies/ml** |  | |  | |  | |  |  | |  |
| **PCV10-type** | 14 (41.2) | | 6 (40.0) | | 16 (55.2) | | 1 (25.0) | 0.87 (0.37, 2.05) | | 0.94 (0.31, 2.87) |
| **Non PCV10-type** | 12 (35.3) | | 8 (53.3) | | 11 (37.9) | | 1 (25.0) | 1.44 (0.55, 3.78) | | 2.17 (0.72, 6.54) |
| ***H. influenzae*** |  | |  | |  | |  |  | |  |
| ***H. influenzae* not type b** | 190 (40.2) | | 91 (47.2) | | 198 (37.4) | | 26 (39.4) | 1.11 (0.82, 1.49) | | **1.56 (1.04, 2.35)** |
| **> 5.9 log_10_ copies/ml** | 71 (15.0) | | 38 (19.7) | | 60 (11.3) | | 7 (10.6) | 1.19 (0.77, 1.84) | | 1.70 (0.99, 2.94) |
| ***H. influenzae* type b** | 25 (5.3) | | 10 (5.2) | | 17 (3.2) | | 8 (12.1) | **2.24 (1.10, 4.58)** | | 2.25 (0.89, 5.68) |
| **>5.9 log_10_ copies/ml** | 9 (1.9) | | 5 (2.6) | | 7 (1.3) | | 5 (7.6) | 1.69 (0.52, 5.45) | | 3.33 (0.87, 12.71) |
| ***S. aureus*** | 78 (16.5) | | 32 (16.6) | | 63 (11.9) | | 15 (22.7) | 1.18 (0.78, 1.80) | | 1.22 (0.69, 2.17) |
| ***B. pertussis*** | 5 (1.1) | | 3 (1.6) | | 0 (0.0) | | 1 (1.5) | --^a^ | | --^a^ |
| ***C. pneumoniae*** | 2 (0.4) | | 0 (0.0) | | 3 (0.6) | | 0 (0.0) | 0.48 (0.05, 4.82) | | --^a^ |
| ***M. catarrhalis*** | 331 (70.0) | | 133 (68.9) | | 418 (78.9) | | 46 (69.7) | 0.56 (0.40, 0.78) | | 0.58 (0.36, 0.93) |
| ***M. pneumoniae*** | 1 (0.2) | | 0 (0.0) | | 0 (0.0) | | 0 (0.0) | --^a^ | | --^a^ |
| **Salmonella species** | 4 (0.8) | | 2 (1.0) | | 0 (0.0) | | 2 (3.0) | --^a^ | | --^a^ |
| **Legionella** | 1 (0.2) | | 0 (0.0) | | 0 (0.0) | | 0 (0.0) | --^a^ | | --^a^ |
| **Fungi** | | | | | | | | | | |
| ***P. jirovecii*** | | 53 (11.2) | 25 (13.0) | 56 (10.6) | | | 15 (22.7) | 1.08 (0.68, 1.70) | 1.28 (0.70, 2.35) | |
| **>4 log_10_ copies/ml** | | 24 (5.1) | 12 (6.2) | 9 (1.7) | | | 10 (15.2) | **3.45 (1.49, 7.98)** | **5.00 (1.90, 13.15)** | |
| **Virus** | | | | | | | | | | |
| **Any virus** | 404 (85.4) | | 165 (85.5) | | 402 (75.8) | | 57 (86.4) | 1.22 (0.79, 1.89) | | 1.21 (0.65, 2.25) |
| **Any virus, with thresholds applied for CMV** | 362 (76.5) | | 150 (77.7) | | 304 (57.4) | | 48 (72.7) | 1.56 (0.94, 2.59) | | 1.75 (0.89, 3.46) |
| **Adenovirus** | 35 (7.4) | | 14 (7.3) | | 31 (5.8) | | 5 (7.6) | 1.59 (0.90, 2.79) | | 1.91 (0.89, 4.11) |
| **CMV** | 247 (52.2) | | 100 (51.8) | | 308 (58.1) | | 43 (65.2) | 0.92 (0.69, 1.23) | | 0.85 (0.56, 1.27) |
| **> 4.9 log10 copies/ml** | 103 (21.8) | | 46 (23.8) | | 109 (20.6) | | 21 (31.8) | 0.91 (0.63, 1.29) | | 0.90 (0.56, 1.47) |
| **Coronavirus 43** | 9 (1.9) | | 4 (2.1) | | 12 (2.3) | | 1 (1.5) | 0.94 (0.34, 2.63) | | 1.15 (0.30, 4.30) |
| **Coronavirus 63** | 9 (1.9) | | 3 (1.6) | | 25 (4.7) | | 1 (1.5) | 0.41 (0.16, 1.00) | | 0.40 (0.10, 1.57) |
| **Coronavirus HKU** | 7 (1.5) | | 4 (2.1) | | 17 (3.2) | | 0 (0.0) | 0.50 (0.18, 1.40) | | 0.60 (0.14, 2.65) |
| **Coronavirus 229** | 4 (0.8) | | 3 (1.6) | | 3 (0.6) | | 1 (1.5) | 1.61 (0.25, 10.29) | | 2.14 (0.21, 21.32) |
| **HBOV** | 60 (12.7) | | 28 (14.5) | | 51 (9.6) | | 6 (9.1) | **1.86 (1.19, 2.89)** | | 1.75 (0.97, 3.15) |
| **HMPV A/B** | 41 (8.7) | | 26 (13.5) | | 10 (1.9) | | 2 (3.0) | **8.02 (3.84, 16.75)** | | **14.51 (6.42, 32.84)** |
| **Influenza A** | 23 (4.9) | | 11 (5.7) | | 7 (1.3) | | 5 (7.6) | **5.60 (2.22, 14.13)** | | **5.22 (1.68, 16.19)** |
| **Influenza B** | 1 (0.2) | | 1 (0.5) | | 0 (0.0) | | 0 (0.0) | --^a^ | | --^a^ |
| **Influenza C** | 1 (0.2) | | 0 (0.0) | | 3 (0.6) | | 0 (0.0) | 0.00 (0.00, I) | | --^a^ |
| **Parainfluenza 1** | 5 (1.1) | | 1 (0.5) | | 1 (0.2) | | 1 (1.5) | 955E9 (0.00, I) | | 3.54E7 (0.00, I) |
| **Parainfluenza 2** | 1 (0.2) | | 0 (0.0) | | 0 (0.0) | | 1 (1.5) | --^a^ | | --^a^ |
| **Parainfluenza 3** | 25 (5.3) | | 11 (5.7) | | 14 (2.6) | | 3 (4.5) | **2.83 (1.38, 5.84)** | | **2.99 (1.17, 7.63)** |
| **Parainfluenza 4** | 10 (2.1) | | 5 (2.6) | | 8 (1.5) | | 0 (0.0) | 1.96 (0.72, 5.36) | | 2.59 (0.72, 9.24) |
| **PV/EV** | 29 (6.1) | | 6 (3.1) | | 35 (6.6) | | 5 (7.6) | 1.13 (0.62, 2.06) | | 0.35 (0.11, 1.07) |
| **Rhinovirus** | 94 (19.9) | | 32 (16.6) | | 93 (17.5) | | 11 (16.7) | **1.91 (1.33, 2.74)** | | 1.45 (0.86, 2.45) |
| **RSV** | 99 (20.9) | | 41 (21.2) | | 18 (3.4) | | 5 (7.6) | **10.26 (5.96, 17.68)** | | **10.75 (5.73, 20.15)** |
| **Whole Blood PCR** |  | |  | |  | |  |  | |  |
| *S. pneumoniae* | 30 (5.8) | | 18 (8.7) | | 24 (4.0) | | 5 (7.9) | 1.55 (0.89, 2.69) | | **2.31 (1.22, 4.37)** |
| >2.2 log10 copies/ml | 19 (3.7) | | 11 (5.3) | | 14 (2.3) | | 4 (6.4) | 1.68 (0.83, 3.39) | | **2.39 (1.07, 5.38)** |

Abbreviations: HBOV, Human bocavirus; CMV, cytomegalovirus; CXR, chest x-ray; HMPV, Human metapneumovirus A/B; NP/OP, nasopharyngeal/oropharyngeal; PCV, pneumococcal conjugate vaccine; PV/EV, Parechovirus/Enterovirus; RSV, Respiratory syncytial virus A/B.

NP/OP PCR odds ratio adjusted for age in months, site, and all other pathogens detected on NP/OP PCR. Whole blood PCR odds ratios adjusted for age in months and site. Bold indicates p < 0.05.

a. Odd ratios could not be calculated due to zero cells.
