# Supplementary material for: The Etiology of Pneumonia in Zambian Children: Findings From the Pneumonia Etiology Research for Child Health (PERCH) Study
Source: Pediatr Infect Dis J. 2021 Aug 25;40(9):S40–9. doi: 10.1097/INF.0000000000002652 (PMC8448410; doi:10.1097/INF.0000000000002652)
Supplement: Supplementary file 7 [file inf-40-s40-s007.docx]

**Supplemental Digital Content 7, Table: Codetection of Organisms in NP/OP Specimens Collected from both HIV-uninfected Cases with severe and very severe pneumonia and Controls**

|  | **All Cases N=473** | **CXR+ Cases N=193** | **All Controls N=530** | **All Cases vs All Controls** | **CXR+ Cases vs All Controls** |
| --- | --- | --- | --- | --- | --- |
| Mean (SD) number of organisms, any positivity | 3.71 (1.56) | 3.81 (1.63) | 3.43 (1.42) |  |  |
| Median (IQR) | 4.0 (3.0, 5.0) | 4.0 (3.0, 5.0) | 4.0 (2.0, 4.0) |  |  |
| 0 | 11 (2.3) | 5 (2.6) | 19 (3.6) | 0.0897 | 0.3391 |
| 1 | 27 (5.7) | 11 (5.7) | 23 (4.3) |  |  |
| 2 | 59 (12.5) | 23 (11.9) | 94 (17.7) |  |  |
| 3 | 116 (24.5) | 42 (21.8) | 117 (22.1) |  |  |
| 4 | 260 (55.0) | 112 (58.0) | 277 (52.3) |  |  |
| Mean (SD) number of organisms, above threshold | 2.38 (1.32) | 2.49 (1.35) | 1.95 (1.18) |  |  |
| Median (IQR) | 2.0 (1.0, 3.0) | 2.0 (2.0, 3.0) | 2.0 (1.0, 3.0) |  |  |
| 0 | 28 (5.9) | 12 (6.2) | 44 (8.3) | **<.0001** | **<.0001** |
| 1 | 103 (21.8) | 35 (18.1) | 163 (30.8) |  |  |
| 2 | 135 (28.5) | 54 (28.0) | 170 (32.1) |  |  |
| 3 | 109 (23.0) | 45 (23.3) | 99 (18.7) |  |  |
| 4 | 98 (20.7) | 47 (24.4) | 54 (10.2) |  |  |
| Pathogen patterns, any positivity |  |  |  |  |  |
| Single bacteria | 13 (2.7) | 6 (3.1) | 14 (2.6) | **0.0012** | 0.0683 |
| 2 or more bacteria | 45 (9.5) | 17 (8.8) | 95 (17.9) |  |  |
| Single virus | 14 (3.0) | 5 (2.6) | 9 (1.7) |  |  |
| 2 or more viruses | 9 (1.9) | 2 (1.0) | 2 (0.4) |  |  |
| Bacterial-Viral | 381 (80.5) | 158 (81.9) | 391 (73.8) |  |  |
| Pathogen patterns, above threshold |  |  |  |  |  |
| Single bacteria | 60 (12.7) | 21 (10.9) | 138 (26.0) | **<.0001** | **<.0001** |
| 2 or more bacteria | 23 (4.9) | 10 (5.2) | 44 (8.3) |  |  |
| Single virus | 43 (9.1) | 14 (7.3) | 25 (4.7) |  |  |
| 2 or more viruses | 23 (4.9) | 9 (4.7) | 6 (1.1) |  |  |
| Bacterial-Viral | 296 (62.6) | 127 (65.8) | 273 (51.5) |  |  |

NP/OP, nasopharyngeal/oropharyngeal. P-values from logistic regression model adjusted for age in months. For above threshold rows, prevalence defined using NP/OP PCR density thresholds for 4 pathogens: *P. jirovecii*, 4 log_10_ copies/mL; *H. influenzae*, 5.9 log_10_ copies/mL; CMV, 4.9 log_10_ copies/mL; *S. pneumoniae*, 6.9 log_10_ copies/mL.
