# Supplementary material for: The Etiology of Pneumonia in Zambian Children: Findings From the Pneumonia Etiology Research for Child Health (PERCH) Study
Source: Pediatr Infect Dis J. 2021 Aug 25;40(9):S40–9. doi: 10.1097/INF.0000000000002652 (PMC8448410; doi:10.1097/INF.0000000000002652)
Supplement: Supplementary file 8 [file inf-40-s40-s008.docx]

**Supplemental Digital Content 8.** **Integrated etiology results of HIV-uninfected Cases with severe and very severe pneumonia**

|  | **Etiologic Fraction (95% CrI)** | | | | | |
| --- | --- | --- | --- | --- | --- | --- |
|  | **All HIV- Cases** | **CXR+ Cases** | | | | |
|  |  |  |  |  |  |  |
| **Etiology** |  | **All CXR+** | **Age < 1** | **Age > 1** | **Severe** | **Very Severe** |
| **Bacteria** |  |  |  |  |  |  |
| *B. pertussis* | 1.3 (0.0, 3.0) | 2.0 (0.0, 5.3) | 2.6 (0.0, 7.0) | 0.3 (0.0, 3.4) | 0.6 (0.0, 3.0) | 4.1 (0.0, 10.0) |
| *C. pneumoniae* | 0.1 (0.0, 0.5) | 0.1 (0.0, 1.1) | 0.1 (0.0, 0.9) | 0.3 (0.0, 3.0) | 0.0 (0.0, 0.7) | 0.1 (0.0, 1.4) |
| *E. coli* | 6.4 (1.2, 16.6) | 6.3 (0.7, 17.7) | 8.2 (0.9, 23.6) | 1.0 (0.0, 10.0) | 10.7 (1.5, 28.1) | 0.6 (0.0, 7.1) |
| *H. influenzae* | 9.5 (3.5, 18.5) | 5.8 (0.4, 18.5) | 6.3 (0.3, 22.8) | 4.5 (0.0, 25.6) | 6.2 (0.7, 18.5) | 4.9 (0.0, 22.9) |
| Type b | 2.7 (0.5, 6.5) | 3.1 (0.2, 9.5) | 3.0 (0.1, 9.7) | 3.4 (0.0, 22.4) | 4.9 (0.7, 14.8) | 2.9 (0.0, 15.7) |
| Non-b | 6.8 (1.6, 15.4) | 2.7 (0.0, 14.7) | 3.2 (0.0, 19.7) | 1.2 (0.0, 12.1) | 1.2 (0.0, 11.1) | 2.0 (0.0, 17.1) |
| Legionella species | 0.2 (0.0, 1.0) | 0.2 (0.0, 1.2) | 0.1 (0.0, 0.9) | 0.3 (0.0, 3.1) | 0.0 (0.0, 0.7) | 0.1 (0.0, 1.4) |
| *M. catarrhalis* | 0.9 (0.0, 6.7) | 1.0 (0.0, 7.0) | 0.6 (0.0, 6.4) | 2.0 (0.0, 19.0) | 0.9 (0.0, 9.6) | 1.3 (0.0, 12.9) |
| *M. pneumoniae* | 0.1 (0.0, 1.0) | 0.2 (0.0, 1.3) | 0.1 (0.0, 0.9) | 0.4 (0.0, 3.6) | 0.0 (0.0, 0.7) | 0.1 (0.0, 1.4) |
| *M. tuberculosis* | 7.3 (2.2, 16.0) | 12.8 (4.3, 25.3) | 17.1 (5.7, 33.8) | 0.9 (0.0, 9.1) | 12.3 (3.7, 27.4) | 15.8 (4.3, 35.7) |
| *N. meningitidis* | 0.5 (0.0, 3.9) | 0.8 (0.0, 6.1) | 0.6 (0.0, 5.8) | 1.4 (0.0, 14.0) | 0.5 (0.0, 5.9) | 0.6 (0.0, 7.1) |
| Non-fermenting gram-negative rods | 0.7 (0.0, 5.4) | 1.0 (0.0, 7.1) | 0.7 (0.0, 7.3) | 1.8 (0.0, 16.9) | 0.9 (0.0, 8.1) | 1.1 (0.0, 11.4) |
| Other streptococci and enterococci | 0.7 (0.0, 4.7) | 1.1 (0.0, 8.1) | 0.9 (0.0, 8.4) | 1.9 (0.0, 17.7) | 0.9 (0.0, 8.9) | 1.2 (0.0, 12.9) |
| *S. aureus* | 6.3 (2.3, 12.2) | 3.6 (0.2, 10.1) | 4.2 (0.2, 12.4) | 2.1 (0.0, 13.5) | 1.4 (0.0, 9.6) | 5.9 (1.4, 15.7) |
| *S. pneumoniae* | 2.9 (0.7, 6.9) | 2.3 (0.1, 7.3) | 0.9 (0.0, 6.2) | 6.3 (0.3, 20.5) | 3.1 (0.7, 10.4) | 1.5 (0.0, 10.0) |
| PCV10 VT | 2.7 (0.6, 6.4) | 1.7 (0.1, 5.6) | 0.3 (0.0, 3.1) | 5.5 (0.2, 18.7) | 2.4 (0.7, 8.1) | 0.9 (0.0, 8.6) |
| PCV10 NVT | 0.2 (0.0, 1.7) | 0.6 (0.0, 4.3) | 0.5 (0.0, 5.1) | 0.8 (0.0, 7.7) | 0.7 (0.0, 5.9) | 0.6 (0.0, 5.7) |
| Salmonella species | 4.4 (1.5, 10.0) | 4.6 (0.8, 12.8) | 3.9 (0.4, 12.5) | 6.5 (0.2, 23.4) | 4.9 (1.5, 14.8) | 4.3 (1.4, 14.3) |
| **Fungi** |  |  |  |  |  |  |
| *Candida* species | 5.3 (0.2, 16.9) | 1.0 (0.0, 7.0) | 0.7 (0.0, 7.5) | 1.7 (0.0, 15.3) | 1.0 (0.0, 10.4) | 1.0 (0.0, 10.0) |
| *P. jirovecii* | 1.4 (0.0, 4.9) | 4.5 (0.0, 9.6) | 6 (0.0, 13.0) | 0.3 (0.0, 2.7) | 3.3 (0.0, 8.1) | 3.8 (0.0, 11.4) |
| **Viruses** |  |  |  |  |  |  |
| Adenovirus | 0.7 (0.0, 3.6) | 0.7 (0.0, 4.3) | 0.6 (0.0, 4.9) | 0.9 (0.0, 8.6) | 0.2 (0.0, 2.2) | 3.0 (0.0, 14.3) |
| Bocavirus | 1.9 (0.0, 6.9) | 0.9 (0.0, 5.6) | 0.8 (0.0, 6.8) | 1.0 (0.0, 8.8) | 0.3 (0.0, 3.7) | 2.2 (0.0, 12.9) |
| CMV | 0.3 (0.0, 1.8) | 0.5 (0.0, 3.6) | 0.4 (0.0, 4.2) | 0.7 (0.0, 6.1) | 0.8 (0.0, 7.4) | 0.4 (0.0, 4.3) |
| Coronavirus | 0.2 (0.0, 1.4) | 0.3 (0.0, 2.0) | 0.2 (0.0, 2.0) | 0.4 (0.0, 4.1) | 0.2 (0.0, 2.2) | 0.1 (0.0, 1.4) |
| HMPV A/B | 7.4 (3.8, 12.3) | 12.8 (6.1, 21.8) | 12.2 (5.4, 22.0) | 14.3 (0.0, 32.4) | 14.3 (8.1, 23.7) | 11.0 (0.0, 21.4) |
| Influenza | 2.9 (0.2, 6.4) | 5.1 (0.7, 10.8) | 1.6 (0.0, 6.8) | 14.7 (0.3, 32.1) | 2.6 (0.0, 8.1) | 6.3 (0.0, 15.7) |
| A | 2.7 (0.0, 6.1) | 4.4 (0.2, 9.9) | 1.0 (0.0, 5.7) | 14.1 (0.0, 31.4) | 2.5 (0.0, 8.1) | 4.6 (0.0, 14.3) |
| B | 0.2 (0.0, 0.9) | 0.5 (0.0, 2.4) | 0.5 (0.0, 3.0) | 0.3 (0.0, 3.3) | 0.0 (0.0, 0.7) | 1.6 (0.0, 5.7) |
| C | 0.1 (0.0, 0.6) | 0.2 (0.0, 1.1) | 0.1 (0.0, 0.9) | 0.3 (0.0, 3.0) | 0.0 (0.0, 0.7) | 0.1 (0.0, 1.4) |
| Parainfluenza | 3 (0.2, 7.2) | 3.1 (0.1, 9.2) | 3.2 (0.0, 10.9) | 2.8 (0.0, 13.4) | 4.2 (0.0, 11.1) | 2.0 (0.0, 8.6) |
| 1 | 0.5 (0.0, 2.1) | 0.2 (0.0, 1.5) | 0.2 (0.0, 1.6) | 0.3 (0.0, 3.2) | 0.0 (0.0, 0.7) | 0.7 (0.0, 4.3) |
| 2 | 0.1 (0.0, 0.8) | 0.2 (0.0, 1.2) | 0.1 (0.0, 0.9) | 0.3 (0.0, 3.3) | 0.0 (0.0, 0.7) | 0.1 (0.0, 1.4) |
| 3 | 1.8 (0.0, 5.4) | 1.9 (0.0, 7.2) | 2.2 (0.0, 9.2) | 1.0 (0.0, 8.0) | 3.2 (0.0, 9.6) | 0.6 (0.0, 5.7) |
| 4 | 0.6 (0.0, 2.7) | 0.9 (0.0, 4.2) | 0.8 (0.0, 4.9) | 1.2 (0.0, 8.5) | 1.0 (0.0, 4.4) | 0.7 (0.0, 5.7) |
| PV/EV | 1.1 (0.0, 4.4) | 0.5 (0.0, 3.0) | 0.2 (0.0, 1.7) | 1.2 (0.0, 10.0) | 0.1 (0.0, 1.5) | 0.7 (0.0, 5.7) |
| Rhinovirus | 7.8 (2.7, 15.7) | 1.0 (0.0, 5.8) | 0.5 (0.0, 4.8) | 2.5 (0.0, 17.3) | 0.6 (0.0, 5.2) | 2.7 (0.0, 17.1) |
| RSV A/B | 24 (17.3, 33.3) | 26.1 (17.0, 37.7) | 25.8 (15.8, 38.4) | 27.0 (10.5, 47.4) | 28.4 (20.0, 40.7) | 23.9 (12.9, 37.1) |
| Not otherwise specified | 2.7 (0.0, 16.5) | 1.8 (0.0, 10.9) | 1.4 (0.0, 13.2) | 2.7 (0.0, 23.5) | 1.6 (0.0, 15.6) | 1.5 (0.0, 14.3) |
| **Summary Estimates** |  |  |  |  |  |  |
| Bacteria* | 34.0 (22.4, 46.5) | 29.0 (15.5, 44.3) | 29.1 (13.8, 47.1) | 28.9 (7.8, 58.2) | 30.0 (14.1, 47.4) | 25.8 (10.0, 47.1) |
| Viruses | 49.3 (38.0, 61.9) | 50.9 (37.8, 64.6) | 45.7 (31.5, 61.5) | 65.6 (36.8, 88.7) | 51.7 (37.0, 67.4) | 52.2 (34.3, 72.9) |

Abbreviation: CMV, cytomegalovirus, CrI, credible interval, HMPV, Human metapneumovirus A/B; PCV10 VT, pneumococcal conjugate vaccine, 10-valent vaccine serotype, PV/EV, parechovirus/enterovirus; RSV, respiratory syncytial virus;

*The bacteria summary estimate excludes *M. tuberculosis*.

CXR+ defined as consolidation and/or other infiltrate on chest radiograph.

For pathogens presented grouped in Figure 2 (e.g., Parainfluenza virus types 1, 2, 3 and 4), both grouped and subspecies level results are presented here, with subspecies level results in gray.

Not Otherwise Specified represents pathogens not tested for. Other Strep includes *Streptococcus pyogenes* and *Enterococcus faecium*. Nonfermentative gram-negative rods includes Acinetobacter species and Pseudomonas species. Enterobacteriaceae includes *E. coli*, Enterobacter species, and Klebsiella species, excluding mixed gram-negative rods.
