# Supplementary material for: The Etiology of Pneumonia in Zambian Children: Findings From the Pneumonia Etiology Research for Child Health (PERCH) Study
Source: Pediatr Infect Dis J. 2021 Aug 25;40(9):S40–9. doi: 10.1097/INF.0000000000002652 (PMC8448410; doi:10.1097/INF.0000000000002652)
Supplement: Supplementary file 10 [file inf-40-s40-s010.docx]

**Supplemental Digital Content 10, Table. Pneumonia etiologic fraction for top ten pathogens among HIV-uninfected cases with positive finding on chest radiograph, stratified by age**

|  | **Overall** | | **Under 1** | | **Over 1** | |
| --- | --- | --- | --- | --- | --- | --- |
| **Rank** | **Pathogen** | **Mean (95% CrI)** | **Pathogen** | **Mean (95% CrI)** | **Pathogen** | **Mean (95% CrI)** |
| 1 | RSV | 26.1 (17.0, 37.7) | **RSV** | 25.8 (15.8, 38.4) | **RSV** | 27.0 (10.5, 47.4) |
| 2 | *M. tuberculosis* | 12.8 (4.3, 25.3) | *M. tuberculosis* | 17.1 (5.7, 33.8) | Influenza | 14.7 (0.3, 32.1) |
| 3 | HMPV A/B | 12.8 (6.1, 21.8) | **HMPV A/B** | 12.2 (5.4, 22.0) | **HMPV A/B** | 14.3 (0, 32.4) |
| 4 | *E. coli* | 6.3 (0.7, 17.7) | *E. coli* | 8.2 (0.9, 23.6) | **Salmonella species** | 6.5 (0.2, 23.4) |
| 5 | *H. influenzae* | 5.8 (0.4, 18.5) | ***H. influenzae*** | 6.3 (0.3, 22.8) | *S. pneumoniae* | 6.3 (0.3, 20.5) |
| 6 | Influenza | 5.1 (0.7, 10.8) | *P. jirovecii* | 6 (0.0, 13.0) | ***H. influenzae*** | 4.5 (0.0, 25.6) |
| 7 | Salmonella species | 4.6 (0.8, 12.8) | ***S. aureus*** | 4.2 (0.2, 12.4) | **Parainfluenza** | 2.8 (0.0, 13.4) |
| 8 | *P. jirovecii* | 4.5 (0.0, 9.6) | **Salmonella species** | 3.9 (0.4, 12.5) | NoS | 2.7 (0.0, 23.5) |
| 9 | *S. aureus* | 3.6 (0.2, 10.1) | **Parainfluenza** | 3.2 (0.0, 10.9) | Rhinovirus | 2.5 (0.0, 17.3) |
| 10 | Parainfluenza | 3.1 (0.1, 9.2) | *B. pertussis* | 2.6 (0.0, 7.0) | ***S. aureus*** | 2.1 (0.0, 13.5) |
| 11 |  |  |  |  | *M. catarrhalis* | 2.0 (0.0, 19) |
|  | Potentially treatable causes^a^ | 37.6 (24.2, 51.2) |  |  |  |  |

Bold=top 10 in both strata. Abbreviations: CrI, credible interval; HMPV, Human metapneumovirus A/B; RSV, Respiratory syncytial virus A/B; NoS, Not Otherwise Specified (i.e., pathogens not tested for).

Pathogens estimated at the subspecies level but grouped to the species level (Parainfluenza types 1, 2, 3 and 4; *S. pneumoniae* PCV 10 and *S. pneumoniae* non PCV 10 types; *H. influenzae* type b and *H. influenzae* non-type b; influenza A, B, C). See Supplemental Digital Content 8 for full list of pathogens including sub-species.

a. Includes *M. tuberculosis*, *E. coli*, *H. influenzae*, *Salmonella*, *P. jirovecii*, and *S. aureus*
